# Supplementary material for: Hot spot prediction in protein-protein interactions by an ensemble system
Source: BMC Syst Biol. 2018 Dec 31;12(Suppl 9):132. doi: 10.1186/s12918-018-0665-8 (PMC6311905; doi:10.1186/s12918-018-0665-8)
Supplement: Supplementary file 2 — All relevant MATLAB code. A simple Matlab implement of our predictor. (ZIP 19,742 kb) [file 12918_2018_665_MOESM2_ESM.zip › Additional file 2/Read ASA/ASA website/dbMPIKT/4GNKB.html]

 NetSurfP - Protein Surface Accessibility and Secondary Structure Predictions

  

|  |  |  |
| --- | --- | --- |
|  |  | NetSurfP - Protein Surface Accessibility and Secondary Structure Predictions  Technical University of Denmark |

  


---

```
# For publication of results, please cite:
# A generic method for assignment of reliability scores applied to solvent accessibility predictions.
# Bent Petersen, Thomas Nordahl Petersen, Pernille Andersen, Morten Nielsen and Claus Lundegaard
# BMC Structural Biology 2009, 9:51 doi:10.1186/1472-6807-9-51
#
# Column 1: Class assignment - B for buried or E for Exposed - Threshold: 25% exposure, but not based on RSA
# Column 2: Amino acid
# Column 3: Sequence name
# Column 4: Amino acid number
# Column 5: Relative Surface Accessibility - RSA
# Column 6: Absolute Surface Accessibility
# Column 7: Z-fit score for RSA prediction
# Column 8: Probability for Alpha-Helix
# Column 9: Probability for Beta-strand
# Column 10: Probability for Coil
E M  4GNKB                  1    0.705 141.151  -1.005   0.003   0.003   0.994
E A  4GNKB                  2    0.432  47.628  -2.089   0.018   0.047   0.935
E H  4GNKB                  3    0.557 101.264  -2.451   0.018   0.088   0.893
E H  4GNKB                  4    0.536  97.480  -1.680   0.019   0.141   0.840
E H  4GNKB                  5    0.516  93.860  -1.007   0.018   0.088   0.893
E H  4GNKB                  6    0.455  82.855  -0.976   0.018   0.088   0.893
E H  4GNKB                  7    0.359  65.357  -1.219   0.019   0.141   0.840
E H  4GNKB                  8    0.485  88.222  -1.235   0.052   0.084   0.864
B G  4GNKB                  9    0.301  23.665  -1.916   0.056   0.142   0.802
E T  4GNKB                 10    0.455  63.039  -0.413   0.056   0.142   0.802
E A  4GNKB                 11    0.385  42.460  -0.643   0.064   0.216   0.721
B L  4GNKB                 12    0.283  51.909  -0.637   0.064   0.216   0.721
E Q  4GNKB                 13    0.370  66.118  -0.394   0.064   0.216   0.721
B L  4GNKB                 14    0.101  18.511  -0.594   0.018   0.088   0.893
E E  4GNKB                 15    0.555  96.958  -0.018   0.018   0.047   0.935
E P  4GNKB                 16    0.486  68.963  -0.182   0.018   0.019   0.964
E P  4GNKB                 17    0.411  58.378  -0.721   0.113   0.043   0.844
E T  4GNKB                 18    0.647  89.781   0.525   0.113   0.043   0.844
B V  4GNKB                 19    0.064   9.775   1.077   0.522   0.016   0.462
E V  4GNKB                 20    0.260  39.916   1.524   0.858   0.002   0.139
E E  4GNKB                 21    0.530  92.661   1.305   0.879   0.010   0.111
B T  4GNKB                 22    0.220  30.556   1.298   0.802   0.014   0.185
B L  4GNKB                 23    0.037   6.775   0.340   0.802   0.014   0.185
E R  4GNKB                 24    0.430  98.378   0.797   0.660   0.049   0.291
E R  4GNKB                 25    0.562 128.790   0.989   0.184   0.043   0.773
E G  4GNKB                 26    0.340  26.797  -0.764   0.018   0.088   0.893
B S  4GNKB                 27    0.070   8.192   0.020   0.005   0.262   0.733
E K  4GNKB                 28    0.393  80.820   1.111   0.001   0.900   0.099
B F  4GNKB                 29    0.032   6.463   0.226   0.001   0.900   0.099
B I  4GNKB                 30    0.203  37.499   1.532   0.001   0.959   0.040
B K  4GNKB                 31    0.162  33.221   0.607   0.001   0.959   0.040
B W  4GNKB                 32    0.099  23.785   0.909   0.001   0.959   0.040
E D  4GNKB                 33    0.270  38.893   0.930   0.003   0.718   0.279
E E  4GNKB                 34    0.662 115.616   0.668   0.005   0.336   0.660
E E  4GNKB                 35    0.778 135.864  -0.097   0.005   0.045   0.951
E T  4GNKB                 36    0.617  85.564  -0.652   0.005   0.015   0.979
E S  4GNKB                 37    0.542  63.487   0.020   0.004   0.138   0.858
E S  4GNKB                 38    0.356  41.700   0.819   0.004   0.616   0.381
E R  4GNKB                 39    0.347  79.440   0.436   0.002   0.816   0.182
B N  4GNKB                 40    0.275  40.333   0.396   0.001   0.900   0.099
E L  4GNKB                 41    0.306  56.010   1.051   0.001   0.959   0.040
B V  4GNKB                 42    0.058   8.915   0.675   0.001   0.959   0.040
B T  4GNKB                 43    0.074  10.236   0.717   0.001   0.959   0.040
B L  4GNKB                 44    0.023   4.211   1.057   0.001   0.959   0.040
B R  4GNKB                 45    0.172  39.480   1.345   0.000   0.983   0.017
B V  4GNKB                 46    0.025   3.873   0.740   0.001   0.959   0.040
B D  4GNKB                 47    0.170  24.468   0.970   0.004   0.514   0.481
E P  4GNKB                 48    0.591  83.835  -0.748   0.005   0.015   0.979
E N  4GNKB                 49    0.595  87.108  -1.159   0.005   0.045   0.951
B G  4GNKB                 50    0.254  19.998  -0.382   0.004   0.138   0.858
B F  4GNKB                 51    0.144  28.861   0.020   0.004   0.420   0.576
B F  4GNKB                 52    0.179  35.885   1.138   0.001   0.959   0.040
B L  4GNKB                 53    0.019   3.461   1.337   0.001   0.959   0.040
B Y  4GNKB                 54    0.150  32.119   1.047   0.001   0.959   0.040
B W  4GNKB                 55    0.051  12.314  -0.274   0.001   0.959   0.040
E T  4GNKB                 56    0.359  49.752   1.103   0.001   0.900   0.099
B G  4GNKB                 57    0.156  12.301  -0.194   0.004   0.514   0.481
E P  4GNKB                 58    0.703  99.727   0.595   0.005   0.045   0.951
E N  4GNKB                 59    0.685 100.299   0.801   0.018   0.019   0.964
E M  4GNKB                 60    0.330  66.073   0.336   0.018   0.047   0.935
E E  4GNKB                 61    0.656 114.621  -0.075   0.021   0.279   0.699
E V  4GNKB                 62    0.554  85.165   0.512   0.004   0.514   0.481
E D  4GNKB                 63    0.283  40.751  -0.278   0.004   0.616   0.381
E T  4GNKB                 64    0.303  42.082   0.861   0.002   0.816   0.182
B L  4GNKB                 65    0.046   8.496   0.272   0.011   0.918   0.071
E D  4GNKB                 66    0.282  40.665   1.029   0.021   0.756   0.223
B I  4GNKB                 67    0.035   6.494  -0.273   0.074   0.484   0.442
E S  4GNKB                 68    0.445  52.131   0.321   0.135   0.317   0.548
E S  4GNKB                 69    0.275  32.195   1.494   0.252   0.423   0.325
B I  4GNKB                 70    0.031   5.735   0.438   0.268   0.505   0.227
E R  4GNKB                 71    0.371  85.005   1.390   0.373   0.401   0.226
E D  4GNKB                 72    0.392  56.430   0.833   0.352   0.332   0.316
B T  4GNKB                 73    0.061   8.530   0.587   0.307   0.165   0.527
B R  4GNKB                 74    0.192  44.083   0.418   0.199   0.152   0.649
E T  4GNKB                 75    0.409  56.756   0.495   0.052   0.084   0.864
B G  4GNKB                 76    0.239  18.809  -2.018   0.053   0.043   0.903
E R  4GNKB                 77    0.540 123.637   0.177   0.053   0.043   0.903
E Y  4GNKB                 78    0.435  93.024  -0.481   0.113   0.087   0.800
B A  4GNKB                 79    0.078   8.596  -2.201   0.113   0.087   0.800
E R  4GNKB                 80    0.546 125.149   0.206   0.191   0.086   0.723
B L  4GNKB                 81    0.229  41.893  -0.524   0.268   0.043   0.689
B P  4GNKB                 82    0.222  31.445  -0.908   0.181   0.016   0.803
E K  4GNKB                 83    0.640 131.730  -0.602   0.181   0.016   0.803
E D  4GNKB                 84    0.508  73.188  -0.613   0.257   0.016   0.727
E P  4GNKB                 85    0.472  66.977  -0.908   0.339   0.016   0.645
E K  4GNKB                 86    0.543 111.675   0.075   0.455   0.046   0.498
B I  4GNKB                 87    0.153  28.286   0.252   0.622   0.015   0.363
E R  4GNKB                 88    0.373  85.348   0.328   0.717   0.014   0.269
E E  4GNKB                 89    0.574 100.313  -0.509   0.717   0.014   0.269
E V  4GNKB                 90    0.288  44.281  -0.665   0.717   0.014   0.269
B L  4GNKB                 91    0.137  25.158  -0.282   0.622   0.015   0.363
E G  4GNKB                 92    0.640  50.352  -0.413   0.522   0.016   0.462
E F  4GNKB                 93    0.424  85.037  -0.888   0.339   0.016   0.645
E G  4GNKB                 94    0.432  34.022  -1.663   0.181   0.016   0.803
E G  4GNKB                 95    0.369  29.009  -1.268   0.115   0.016   0.868
E P  4GNKB                 96    0.635  90.106  -2.042   0.115   0.016   0.868
E D  4GNKB                 97    0.636  91.676  -1.947   0.058   0.017   0.925
E A  4GNKB                 98    0.450  49.601  -1.336   0.113   0.043   0.844
E R  4GNKB                 99    0.468 107.149  -0.065   0.113   0.043   0.844
B L  4GNKB                100    0.181  33.178  -0.061   0.113   0.043   0.844
E E  4GNKB                101    0.436  76.152  -0.235   0.018   0.019   0.964
E E  4GNKB                102    0.521  90.966   0.488   0.005   0.045   0.951
B K  4GNKB                103    0.126  26.000   0.928   0.005   0.262   0.733
B L  4GNKB                104    0.024   4.339   0.877   0.002   0.816   0.182
B M  4GNKB                105    0.020   4.062   1.218   0.001   0.959   0.040
B T  4GNKB                106    0.025   3.454   0.995   0.000   0.983   0.017
B V  4GNKB                107    0.013   2.044   1.855   0.000   0.983   0.017
B V  4GNKB                108    0.023   3.550   0.852   0.001   0.959   0.040
B S  4GNKB                109    0.038   4.418   0.318   0.001   0.959   0.040
B G  4GNKB                110    0.076   5.997  -0.924   0.004   0.616   0.381
E P  4GNKB                111    0.301  42.769  -0.404   0.004   0.138   0.858
E D  4GNKB                112    0.565  81.474  -0.494   0.005   0.045   0.951
E P  4GNKB                113    0.465  65.969  -1.363   0.005   0.045   0.951
E V  4GNKB                114    0.398  61.142  -1.031   0.004   0.085   0.910
E N  4GNKB                115    0.481  70.448  -0.150   0.005   0.262   0.733
B T  4GNKB                116    0.166  23.010   0.597   0.004   0.616   0.381
E V  4GNKB                117    0.325  50.029   0.810   0.002   0.816   0.182
B F  4GNKB                118    0.078  15.695   0.624   0.001   0.900   0.099
B L  4GNKB                119    0.029   5.255   0.816   0.011   0.918   0.071
B N  4GNKB                120    0.109  15.884  -0.044   0.011   0.918   0.071
B F  4GNKB                121    0.029   5.720   0.647   0.018   0.846   0.136
B M  4GNKB                122    0.054  10.785  -0.373   0.003   0.718   0.279
B A  4GNKB                123    0.030   3.295  -0.137   0.004   0.420   0.576
E V  4GNKB                124    0.408  62.725  -0.385   0.005   0.045   0.951
E Q  4GNKB                125    0.307  54.866   0.967   0.003   0.003   0.994
E D  4GNKB                126    0.554  79.903   1.167   0.923   0.002   0.076
E D  4GNKB                127    0.674  97.080   0.681   0.923   0.002   0.076
B T  4GNKB                128    0.102  14.203   0.343   0.970   0.001   0.030
B A  4GNKB                129    0.045   4.915   0.702   0.970   0.001   0.030
E K  4GNKB                130    0.533 109.700   1.710   0.970   0.001   0.030
E V  4GNKB                131    0.277  42.590   2.547   0.970   0.001   0.030
B W  4GNKB                132    0.020   4.834   1.482   0.970   0.001   0.030
B S  4GNKB                133    0.096  11.204   0.633   0.988   0.000   0.012
E E  4GNKB                134    0.400  69.810   1.481   0.988   0.000   0.012
B E  4GNKB                135    0.064  11.251  -0.222   0.988   0.000   0.012
B L  4GNKB                136    0.028   5.072   0.398   0.988   0.000   0.012
E F  4GNKB                137    0.280  56.236   0.843   0.988   0.000   0.012
E K  4GNKB                138    0.286  58.830   1.889   0.988   0.000   0.012
B L  4GNKB                139    0.033   6.079   0.522   0.970   0.001   0.030
B A  4GNKB                140    0.124  13.665   0.798   0.970   0.001   0.030
E M  4GNKB                141    0.451  90.245   0.479   0.970   0.001   0.030
E N  4GNKB                142    0.321  46.921   0.475   0.923   0.002   0.076
B I  4GNKB                143    0.119  21.997  -0.539   0.858   0.002   0.139
E L  4GNKB                144    0.401  73.423  -0.805   0.694   0.003   0.303
E A  4GNKB                145    0.637  70.164  -0.577   0.502   0.002   0.495
E Q  4GNKB                146    0.422  75.423  -1.073   0.176   0.004   0.820
E N  4GNKB                147    0.529  77.431  -1.488   0.109   0.005   0.886
E A  4GNKB                148    0.413  45.513  -0.898   0.176   0.004   0.820
E S  4GNKB                149    0.567  66.452  -0.595   0.339   0.016   0.645
E R  4GNKB                150    0.368  84.364  -0.034   0.802   0.014   0.185
E N  4GNKB                151    0.301  44.022  -0.737   0.923   0.002   0.076
E T  4GNKB                152    0.452  62.692   0.078   0.970   0.001   0.030
E F  4GNKB                153    0.300  60.170  -0.310   0.970   0.001   0.030
B L  4GNKB                154    0.079  14.410  -0.167   0.970   0.001   0.030
E R  4GNKB                155    0.281  64.303   0.543   0.970   0.001   0.030
E K  4GNKB                156    0.372  76.541   0.534   0.970   0.001   0.030
B A  4GNKB                157    0.202  22.271   0.950   0.970   0.001   0.030
B Y  4GNKB                158    0.075  15.985  -0.311   0.970   0.001   0.030
E T  4GNKB                159    0.282  39.072   0.573   0.970   0.001   0.030
E K  4GNKB                160    0.383  78.701   0.240   0.923   0.002   0.076
B L  4GNKB                161    0.188  34.331  -0.729   0.923   0.002   0.076
B K  4GNKB                162    0.223  45.809  -0.194   0.858   0.002   0.139
B L  4GNKB                163    0.255  46.654  -1.048   0.600   0.003   0.397
E Q  4GNKB                164    0.456  81.459  -0.610   0.430   0.016   0.555
E V  4GNKB                165    0.395  60.696  -0.416   0.339   0.016   0.645
E N  4GNKB                166    0.373  54.651  -0.993   0.176   0.004   0.820
E Q  4GNKB                167    0.681 121.662  -1.219   0.053   0.005   0.942
E D  4GNKB                168    0.643  92.671  -0.721   0.016   0.005   0.979
E G  4GNKB                169    0.455  35.848  -0.509   0.005   0.015   0.979
E R  4GNKB                170    0.537 122.904   1.199   0.004   0.085   0.910
B I  4GNKB                171    0.040   7.455   0.293   0.004   0.197   0.799
E P  4GNKB                172    0.347  49.268   1.253   0.004   0.138   0.858
B V  4GNKB                173    0.144  22.087   0.510   0.923   0.002   0.076
E K  4GNKB                174    0.588 120.972   1.253   0.970   0.001   0.030
E N  4GNKB                175    0.309  45.296   1.181   0.970   0.001   0.030
B I  4GNKB                176    0.019   3.423   0.856   0.970   0.001   0.030
B L  4GNKB                177    0.243  44.512   1.189   0.970   0.001   0.030
E K  4GNKB                178    0.497 102.315   1.772   0.923   0.002   0.076
B M  4GNKB                179    0.056  11.226  -0.303   0.923   0.002   0.076
B F  4GNKB                180    0.038   7.586  -0.217   0.858   0.002   0.139
E S  4GNKB                181    0.477  55.928   0.594   0.782   0.003   0.216
E A  4GNKB                182    0.511  56.312  -0.679   0.600   0.003   0.397
E D  4GNKB                183    0.361  52.092  -1.011   0.321   0.003   0.675
E K  4GNKB                184    0.502 103.323  -1.068   0.109   0.005   0.886
B K  4GNKB                185    0.256  52.659  -0.335   0.058   0.017   0.925
E R  4GNKB                186    0.505 115.714   0.194   0.053   0.043   0.903
B V  4GNKB                187    0.057   8.684   0.194   0.199   0.152   0.649
E E  4GNKB                188    0.565  98.775   0.916   0.502   0.102   0.396
B T  4GNKB                189    0.237  32.886   0.170   0.455   0.046   0.498
B A  4GNKB                190    0.055   6.105  -0.420   0.522   0.016   0.462
E L  4GNKB                191    0.280  51.341   0.126   0.923   0.002   0.076
E E  4GNKB                192    0.619 108.122   0.825   0.970   0.001   0.030
E S  4GNKB                193    0.293  34.304   1.229   0.970   0.001   0.030
B C  4GNKB                194    0.016   2.218   1.069   0.923   0.002   0.076
E G  4GNKB                195    0.434  34.164   1.390   0.858   0.002   0.139
E L  4GNKB                196    0.451  82.615   0.758   0.782   0.003   0.216
B K  4GNKB                197    0.171  35.092   0.767   0.782   0.003   0.216
B F  4GNKB                198    0.080  16.136  -0.689   0.694   0.003   0.303
E N  4GNKB                199    0.562  82.306   0.847   0.717   0.014   0.269
E R  4GNKB                200    0.479 109.691   0.927   0.717   0.014   0.269
B S  4GNKB                201    0.095  11.146  -0.523   0.522   0.016   0.462
E E  4GNKB                202    0.387  67.556  -0.372   0.339   0.016   0.645
E S  4GNKB                203    0.517  60.628  -0.423   0.339   0.016   0.645
E I  4GNKB                204    0.462  85.544  -0.066   0.268   0.043   0.689
E R  4GNKB                205    0.566 129.706   0.413   0.058   0.017   0.925
E P  4GNKB                206    0.550  78.031   0.144   0.058   0.017   0.925
E D  4GNKB                207    0.645  93.017  -0.329   0.018   0.047   0.935
E E  4GNKB                208    0.485  84.782   1.151   0.056   0.142   0.802
B F  4GNKB                209    0.056  11.219   0.680   0.018   0.088   0.893
E S  4GNKB                210    0.433  50.794   0.626   0.113   0.043   0.844
B L  4GNKB                211    0.283  51.817   0.629   0.970   0.001   0.030
E E  4GNKB                212    0.686 119.932   0.570   0.970   0.001   0.030
B I  4GNKB                213    0.227  42.050   0.914   0.970   0.001   0.030
B F  4GNKB                214    0.052  10.477   0.467   0.988   0.000   0.012
B E  4GNKB                215    0.215  37.491   1.889   0.988   0.000   0.012
E R  4GNKB                216    0.455 104.081   1.961   0.988   0.000   0.012
B F  4GNKB                217    0.040   7.948   0.486   0.970   0.001   0.030
B L  4GNKB                218    0.052   9.558   0.655   0.970   0.001   0.030
E N  4GNKB                219    0.479  70.155   1.501   0.923   0.002   0.076
E K  4GNKB                220    0.417  85.777   1.433   0.782   0.003   0.216
B L  4GNKB                221    0.077  14.172  -0.865   0.406   0.004   0.590
B C  4GNKB                222    0.250  35.072  -0.043   0.109   0.005   0.886
E L  4GNKB                223    0.561 102.811   0.230   0.053   0.005   0.942
E R  4GNKB                224    0.270  61.830   0.525   0.321   0.003   0.675
E P  4GNKB                225    0.647  91.767   0.114   0.600   0.003   0.397
E D  4GNKB                226    0.323  46.515   0.624   0.782   0.003   0.216
B I  4GNKB                227    0.022   4.089   0.749   0.970   0.001   0.030
E D  4GNKB                228    0.324  46.631   1.582   0.970   0.001   0.030
E K  4GNKB                229    0.485  99.847   1.805   0.970   0.001   0.030
B I  4GNKB                230    0.062  11.451   0.990   0.970   0.001   0.030
B L  4GNKB                231    0.046   8.423   0.369   0.970   0.001   0.030
E L  4GNKB                232    0.481  87.998   1.315   0.970   0.001   0.030
E E  4GNKB                233    0.509  88.975   1.078   0.938   0.007   0.055
B I  4GNKB                234    0.088  16.224   0.586   0.717   0.014   0.269
B G  4GNKB                235    0.252  19.809  -0.710   0.430   0.016   0.555
E A  4GNKB                236    0.477  52.587  -0.769   0.109   0.005   0.886
E K  4GNKB                237    0.679 139.629   0.281   0.053   0.005   0.942
E G  4GNKB                238    0.777  61.126  -1.187   0.016   0.005   0.979
E K  4GNKB                239    0.495 101.739   0.015   0.016   0.005   0.979
E P  4GNKB                240    0.616  87.467   0.368   0.005   0.015   0.979
E Y  4GNKB                241    0.313  66.909   0.626   0.004   0.138   0.858
B L  4GNKB                242    0.037   6.738  -0.137   0.004   0.197   0.799
E T  4GNKB                243    0.310  43.011   1.447   0.005   0.045   0.951
E L  4GNKB                244    0.255  46.709   0.643   0.970   0.001   0.030
E E  4GNKB                245    0.674 117.695   0.551   0.970   0.001   0.030
E Q  4GNKB                246    0.289  51.615   1.214   0.970   0.001   0.030
B L  4GNKB                247    0.026   4.797   0.611   0.970   0.001   0.030
B M  4GNKB                248    0.214  42.901   1.584   0.970   0.001   0.030
E D  4GNKB                249    0.496  71.445   2.124   0.970   0.001   0.030
B F  4GNKB                250    0.077  15.474   0.978   0.988   0.000   0.012
B I  4GNKB                251    0.036   6.678   0.736   0.970   0.001   0.030
E N  4GNKB                252    0.437  64.035   1.699   0.970   0.001   0.030
E Q  4GNKB                253    0.533  95.283   1.005   0.970   0.001   0.030
B K  4GNKB                254    0.239  49.265   1.155   0.879   0.010   0.111
B Q  4GNKB                255    0.240  42.918   0.071   0.522   0.016   0.462
E R  4GNKB                256    0.652 149.216   0.096   0.109   0.005   0.886
E D  4GNKB                257    0.273  39.383   0.309   0.058   0.017   0.925
E P  4GNKB                258    0.665  94.406  -0.470   0.058   0.017   0.925
E R  4GNKB                259    0.507 116.126  -0.069   0.181   0.016   0.803
B L  4GNKB                260    0.346  63.353  -1.226   0.430   0.016   0.555
E N  4GNKB                261    0.507  74.181  -0.828   0.622   0.015   0.363
E E  4GNKB                262    0.501  87.577  -0.520   0.717   0.014   0.269
E V  4GNKB                263    0.389  59.805   0.198   0.622   0.015   0.363
B L  4GNKB                264    0.207  37.938  -0.526   0.622   0.015   0.363
B Y  4GNKB                265    0.265  56.545   0.177   0.622   0.015   0.363
E P  4GNKB                266    0.393  55.696  -0.339   0.622   0.015   0.363
E P  4GNKB                267    0.429  60.833  -0.454   0.622   0.015   0.363
B L  4GNKB                268    0.201  36.803  -0.373   0.430   0.016   0.555
E R  4GNKB                269    0.296  67.853   0.218   0.339   0.016   0.645
E P  4GNKB                270    0.408  57.924   0.570   0.923   0.002   0.076
E S  4GNKB                271    0.654  76.696   0.883   0.970   0.001   0.030
B Q  4GNKB                272    0.219  39.096   1.496   0.970   0.001   0.030
B A  4GNKB                273    0.026   2.810   0.354   0.970   0.001   0.030
E R  4GNKB                274    0.370  84.776   1.465   0.970   0.001   0.030
E L  4GNKB                275    0.456  83.549   1.153   0.970   0.001   0.030
B L  4GNKB                276    0.045   8.258  -0.056   0.970   0.001   0.030
B I  4GNKB                277    0.081  14.966  -0.048   0.923   0.002   0.076
E E  4GNKB                278    0.580 101.309   1.032   0.782   0.003   0.216
E K  4GNKB                279    0.501 103.076   0.591   0.694   0.003   0.303
B Y  4GNKB                280    0.189  40.475   0.002   0.339   0.016   0.645
E E  4GNKB                281    0.460  80.414  -0.651   0.058   0.017   0.925
E P  4GNKB                282    0.495  70.198  -0.684   0.018   0.019   0.964
E N  4GNKB                283    0.487  71.326  -0.963   0.115   0.016   0.868
E Q  4GNKB                284    0.584 104.302  -1.257   0.339   0.016   0.645
E Q  4GNKB                285    0.589 105.195  -0.381   0.354   0.048   0.598
B F  4GNKB                286    0.261  52.363  -1.697   0.354   0.048   0.598
E L  4GNKB                287    0.400  73.313  -0.134   0.354   0.048   0.598
E E  4GNKB                288    0.611 106.742  -1.455   0.268   0.043   0.689
E R  4GNKB                289    0.550 125.904  -0.291   0.053   0.043   0.903
E D  4GNKB                290    0.525  75.624  -1.009   0.018   0.047   0.935
B Q  4GNKB                291    0.257  45.864  -0.044   0.018   0.088   0.893
B M  4GNKB                292    0.052  10.405  -0.023   0.004   0.085   0.910
E S  4GNKB                293    0.243  28.421   0.713   0.018   0.047   0.935
B M  4GNKB                294    0.152  30.355   0.259   0.923   0.002   0.076
E E  4GNKB                295    0.402  70.160  -0.330   0.923   0.002   0.076
B G  4GNKB                296    0.088   6.926  -0.665   0.923   0.002   0.076
B F  4GNKB                297    0.022   4.435   0.598   0.970   0.001   0.030
B S  4GNKB                298    0.095  11.099   0.262   0.970   0.001   0.030
E R  4GNKB                299    0.307  70.303   0.984   0.923   0.002   0.076
B Y  4GNKB                300    0.064  13.634   0.371   0.923   0.002   0.076
B L  4GNKB                301    0.030   5.456  -0.384   0.782   0.003   0.216
B G  4GNKB                302    0.153  12.017  -0.184   0.406   0.004   0.590
B G  4GNKB                303    0.129  10.192   0.206   0.053   0.005   0.942
E E  4GNKB                304    0.609 106.375   0.293   0.053   0.005   0.942
E E  4GNKB                305    0.530  92.591  -1.259   0.018   0.019   0.964
B N  4GNKB                306    0.127  18.520  -1.801   0.018   0.019   0.964
B G  4GNKB                307    0.296  23.335  -1.146   0.018   0.047   0.935
B I  4GNKB                308    0.096  17.797  -2.741   0.018   0.047   0.935
B L  4GNKB                309    0.200  36.712   0.273   0.018   0.047   0.935
E P  4GNKB                310    0.313  44.429   0.378   0.018   0.047   0.935
E L  4GNKB                311    0.487  89.133  -0.819   0.058   0.017   0.925
E E  4GNKB                312    0.687 120.089  -0.510   0.115   0.016   0.868
E A  4GNKB                313    0.350  38.570   0.194   0.113   0.043   0.844
E L  4GNKB                314    0.500  91.605  -0.376   0.184   0.043   0.773
E D  4GNKB                315    0.597  86.013   0.849   0.278   0.093   0.628
B L  4GNKB                316    0.180  33.031  -0.669   0.307   0.165   0.527
E S  4GNKB                317    0.344  40.282  -0.268   0.307   0.165   0.527
E T  4GNKB                318    0.397  55.022  -0.506   0.307   0.165   0.527
E D  4GNKB                319    0.307  44.181  -0.647   0.278   0.093   0.628
B M  4GNKB                320    0.148  29.675   0.098   0.268   0.043   0.689
E T  4GNKB                321    0.528  73.275  -0.342   0.184   0.043   0.773
E Q  4GNKB                322    0.316  56.455   0.527   0.354   0.048   0.598
B P  4GNKB                323    0.222  31.516  -0.842   0.561   0.047   0.393
B L  4GNKB                324    0.073  13.330   0.035   0.561   0.047   0.393
B S  4GNKB                325    0.154  18.002  -0.189   0.561   0.047   0.393
E A  4GNKB                326    0.302  33.280  -0.105   0.561   0.047   0.393
B Y  4GNKB                327    0.127  27.097   0.246   0.538   0.173   0.289
B F  4GNKB                328    0.118  23.663   0.129   0.453   0.248   0.299
B I  4GNKB                329    0.083  15.318  -0.572   0.352   0.332   0.316
E N  4GNKB                330    0.276  40.348  -1.246   0.125   0.227   0.648
B S  4GNKB                331    0.175  20.475  -1.548   0.052   0.084   0.864
B S  4GNKB                332    0.253  29.663  -1.654   0.052   0.084   0.864
B H  4GNKB                333    0.255  46.312  -1.218   0.052   0.084   0.864
B N  4GNKB                334    0.244  35.678  -1.302   0.113   0.087   0.800
B T  4GNKB                335    0.181  25.077  -1.073   0.216   0.235   0.548
B Y  4GNKB                336    0.167  35.688  -0.860   0.135   0.317   0.548
B L  4GNKB                337    0.109  19.885  -0.224   0.148   0.418   0.435
B T  4GNKB                338    0.176  24.383  -0.495   0.135   0.317   0.548
B A  4GNKB                339    0.282  31.054  -1.253   0.125   0.227   0.648
E G  4GNKB                340    0.434  34.164  -1.091   0.056   0.142   0.802
B Q  4GNKB                341    0.220  39.346  -0.597   0.125   0.227   0.648
B L  4GNKB                342    0.124  22.668  -1.377   0.066   0.296   0.638
E A  4GNKB                343    0.417  45.909  -0.365   0.066   0.296   0.638
B G  4GNKB                344    0.184  14.465  -1.414   0.020   0.205   0.775
E T  4GNKB                345    0.490  68.032  -0.314   0.056   0.142   0.802
B S  4GNKB                346    0.149  17.498  -1.596   0.052   0.084   0.864
E S  4GNKB                347    0.300  35.137  -0.175   0.053   0.043   0.903
B V  4GNKB                348    0.109  16.676  -0.980   0.879   0.010   0.111
E E  4GNKB                349    0.419  73.199   0.720   0.923   0.002   0.076
B M  4GNKB                350    0.055  10.965   0.094   0.970   0.001   0.030
B Y  4GNKB                351    0.031   6.539   0.535   0.970   0.001   0.030
B R  4GNKB                352    0.110  25.190   0.158   0.970   0.001   0.030
E Q  4GNKB                353    0.333  59.527   1.233   0.970   0.001   0.030
B A  4GNKB                354    0.025   2.799   0.423   0.970   0.001   0.030
B L  4GNKB                355    0.039   7.232   0.131   0.923   0.002   0.076
E L  4GNKB                356    0.396  72.471   0.360   0.782   0.003   0.216
E W  4GNKB                357    0.402  96.729   0.324   0.321   0.003   0.675
E G  4GNKB                358    0.399  31.362  -1.232   0.018   0.019   0.964
B C  4GNKB                359    0.044   6.150  -0.228   0.005   0.045   0.951
E R  4GNKB                360    0.282  64.509   1.041   0.004   0.420   0.576
B C  4GNKB                361    0.036   4.998  -0.067   0.001   0.900   0.099
B V  4GNKB                362    0.051   7.869   0.836   0.001   0.959   0.040
B E  4GNKB                363    0.105  18.413  -0.211   0.001   0.959   0.040
B L  4GNKB                364    0.037   6.738   0.010   0.001   0.900   0.099
B D  4GNKB                365    0.124  17.840  -0.291   0.003   0.718   0.279
B V  4GNKB                366    0.042   6.440  -0.683   0.004   0.420   0.576
B W  4GNKB                367    0.155  37.278  -0.109   0.004   0.420   0.576
E K  4GNKB                368    0.285  58.522  -0.898   0.020   0.205   0.775
B G  4GNKB                369    0.261  20.564  -2.160   0.018   0.019   0.964
E R  4GNKB                370    0.601 137.560  -1.592   0.018   0.047   0.935
E P  4GNKB                371    0.443  62.805  -1.093   0.018   0.047   0.935
E P  4GNKB                372    0.607  86.077  -1.500   0.018   0.019   0.964
E E  4GNKB                373    0.614 107.301  -2.106   0.005   0.015   0.979
E E  4GNKB                374    0.689 120.386  -1.336   0.005   0.015   0.979
E E  4GNKB                375    0.363  63.486  -0.109   0.004   0.085   0.910
B P  4GNKB                376    0.098  13.949  -0.775   0.005   0.336   0.660
E F  4GNKB                377    0.251  50.295   0.355   0.002   0.816   0.182
B I  4GNKB                378    0.050   9.287  -0.716   0.001   0.900   0.099
B T  4GNKB                379    0.186  25.784  -0.190   0.021   0.756   0.223
B H  4GNKB                380    0.169  30.687  -0.475   0.021   0.279   0.699
B G  4GNKB                381    0.267  21.013  -1.334   0.018   0.019   0.964
E F  4GNKB                382    0.424  85.137  -0.729   0.018   0.088   0.893
E T  4GNKB                383    0.326  45.244  -1.408   0.021   0.279   0.699
B M  4GNKB                384    0.182  36.338  -1.064   0.021   0.279   0.699
B T  4GNKB                385    0.216  29.890  -1.104   0.020   0.205   0.775
E T  4GNKB                386    0.368  50.972  -1.054   0.019   0.141   0.840
E E  4GNKB                387    0.572  99.998  -0.242   0.052   0.084   0.864
B V  4GNKB                388    0.092  14.140   0.210   0.113   0.043   0.844
E P  4GNKB                389    0.313  44.457   0.125   0.191   0.086   0.723
B L  4GNKB                390    0.062  11.407  -1.572   0.802   0.014   0.185
E R  4GNKB                391    0.360  82.532   1.195   0.938   0.007   0.055
E D  4GNKB                392    0.340  49.052   0.760   0.938   0.007   0.055
B V  4GNKB                393    0.033   5.103   0.264   0.975   0.003   0.022
B L  4GNKB                394    0.051   9.375   0.295   0.975   0.003   0.022
E E  4GNKB                395    0.448  78.301   1.672   0.975   0.003   0.022
B A  4GNKB                396    0.056   6.127   0.528   0.975   0.003   0.022
B I  4GNKB                397    0.026   4.810   0.635   0.938   0.007   0.055
E A  4GNKB                398    0.356  39.187   1.128   0.858   0.002   0.139
E E  4GNKB                399    0.552  96.365   0.725   0.782   0.003   0.216
B T  4GNKB                400    0.222  30.791   0.561   0.600   0.003   0.397
B A  4GNKB                401    0.074   8.122  -1.498   0.430   0.016   0.555
B F  4GNKB                402    0.185  37.109  -1.638   0.268   0.043   0.689
E K  4GNKB                403    0.427  87.772  -0.322   0.268   0.043   0.689
E T  4GNKB                404    0.447  61.957  -0.425   0.184   0.043   0.773
B S  4GNKB                405    0.172  20.217  -0.086   0.053   0.043   0.903
E P  4GNKB                406    0.574  81.479   0.120   0.058   0.017   0.925
B Y  4GNKB                407    0.130  27.866   0.318   0.058   0.017   0.925
B P  4GNKB                408    0.070   9.990  -0.110   0.053   0.043   0.903
B V  4GNKB                409    0.039   5.994  -0.333   0.074   0.484   0.442
B I  4GNKB                410    0.057  10.490  -0.129   0.018   0.846   0.136
B L  4GNKB                411    0.046   8.478   0.255   0.072   0.855   0.073
B S  4GNKB                412    0.040   4.735  -0.764   0.085   0.775   0.140
B F  4GNKB                413    0.037   7.466  -0.745   0.079   0.592   0.329
B E  4GNKB                414    0.189  33.071  -0.053   0.148   0.418   0.435
B N  4GNKB                415    0.213  31.183  -1.064   0.056   0.142   0.802
E H  4GNKB                416    0.295  53.751  -0.477   0.018   0.047   0.935
B V  4GNKB                417    0.054   8.300  -1.407   0.018   0.047   0.935
E D  4GNKB                418    0.432  62.194  -0.781   0.005   0.015   0.979
E S  4GNKB                419    0.332  38.910   0.274   0.005   0.015   0.979
E A  4GNKB                420    0.297  32.729   0.094   0.782   0.003   0.216
E K  4GNKB                421    0.648 133.335   1.041   0.923   0.002   0.076
B Q  4GNKB                422    0.212  37.917  -0.089   0.923   0.002   0.076
B Q  4GNKB                423    0.085  15.252   0.139   0.970   0.001   0.030
E A  4GNKB                424    0.431  47.518   1.032   0.970   0.001   0.030
E K  4GNKB                425    0.347  71.296   1.566   0.988   0.000   0.012
B M  4GNKB                426    0.022   4.342   0.792   0.988   0.000   0.012
B A  4GNKB                427    0.033   3.593  -0.153   0.988   0.000   0.012
E E  4GNKB                428    0.507  88.590   1.766   0.988   0.000   0.012
B Y  4GNKB                429    0.122  25.965   1.182   0.988   0.000   0.012
B C  4GNKB                430    0.027   3.735   0.744   0.988   0.000   0.012
E R  4GNKB                431    0.351  80.425   1.339   0.970   0.001   0.030
E S  4GNKB                432    0.646  75.699   1.279   0.970   0.001   0.030
B I  4GNKB                433    0.135  25.049   0.543   0.923   0.002   0.076
B F  4GNKB                434    0.082  16.357   0.080   0.694   0.003   0.303
E G  4GNKB                435    0.391  30.764  -0.196   0.321   0.003   0.675
E D  4GNKB                436    0.485  69.946  -0.824   0.339   0.016   0.645
E A  4GNKB                437    0.280  30.889   0.623   0.278   0.093   0.628
B L  4GNKB                438    0.092  16.827  -0.119   0.199   0.152   0.649
B L  4GNKB                439    0.180  32.976  -0.049   0.125   0.227   0.648
E I  4GNKB                440    0.330  61.069  -0.307   0.056   0.142   0.802
E E  4GNKB                441    0.448  78.266  -0.309   0.018   0.088   0.893
E P  4GNKB                442    0.449  63.770  -1.080   0.018   0.047   0.935
E L  4GNKB                443    0.394  72.178  -1.682   0.018   0.019   0.964
E D  4GNKB                444    0.627  90.336  -1.665   0.058   0.017   0.925
E K  4GNKB                445    0.597 122.885  -2.128   0.115   0.016   0.868
E Y  4GNKB                446    0.385  82.339  -1.293   0.115   0.016   0.868
E P  4GNKB                447    0.522  74.086  -1.454   0.115   0.016   0.868
E L  4GNKB                448    0.412  75.437  -1.259   0.113   0.043   0.844
E A  4GNKB                449    0.536  59.089  -1.241   0.053   0.043   0.903
E P  4GNKB                450    0.606  86.034  -1.530   0.058   0.017   0.925
E G  4GNKB                451    0.476  37.430  -1.664   0.018   0.019   0.964
E V  4GNKB                452    0.542  83.290  -0.679   0.053   0.043   0.903
E P  4GNKB                453    0.497  70.482   0.072   0.018   0.088   0.893
B L  4GNKB                454    0.116  21.203   0.579   0.018   0.088   0.893
B P  4GNKB                455    0.196  27.841  -0.949   0.018   0.047   0.935
E S  4GNKB                456    0.256  29.945   0.231   0.058   0.017   0.925
B P  4GNKB                457    0.169  23.953  -0.681   0.622   0.015   0.363
E Q  4GNKB                458    0.473  84.460  -0.095   0.622   0.015   0.363
E D  4GNKB                459    0.477  68.765   0.682   0.561   0.047   0.393
B L  4GNKB                460    0.076  13.952  -0.297   0.561   0.047   0.393
E M  4GNKB                461    0.433  86.663  -0.123   0.386   0.097   0.517
E G  4GNKB                462    0.443  34.856  -0.266   0.118   0.150   0.732
B R  4GNKB                463    0.267  61.074   1.249   0.074   0.484   0.442
B I  4GNKB                464    0.055  10.194   0.362   0.021   0.756   0.223
B L  4GNKB                465    0.058  10.547   0.319   0.021   0.756   0.223
B V  4GNKB                466    0.109  16.692   0.134   0.023   0.655   0.322
B K  4GNKB                467    0.211  43.506  -0.376   0.022   0.552   0.426
E N  4GNKB                468    0.357  52.236  -1.312   0.021   0.451   0.528
E K  4GNKB                469    0.291  59.838   0.201   0.021   0.451   0.528
E K  4GNKB                470    0.463  95.301  -0.213   0.022   0.359   0.619
B R  4GNKB                471    0.150  34.396  -0.271   0.019   0.141   0.840
E H  4GNKB                472    0.461  83.892  -0.457   0.053   0.043   0.903
E R  4GNKB                473    0.533 122.149  -1.328   0.113   0.043   0.844
E P  4GNKB                474    0.360  51.141  -0.691   0.191   0.086   0.723
E S  4GNKB                475    0.321  37.633  -1.228   0.191   0.086   0.723
E A  4GNKB                476    0.600  66.142  -0.751   0.191   0.086   0.723
E G  4GNKB                477    0.470  36.965  -1.235   0.278   0.093   0.628
B G  4GNKB                478    0.244  19.187  -0.192   0.278   0.093   0.628
E P  4GNKB                479    0.444  63.004  -0.470   0.278   0.093   0.628
E D  4GNKB                480    0.421  60.709  -0.371   0.278   0.093   0.628
E S  4GNKB                481    0.522  61.214  -1.032   0.386   0.097   0.517
E A  4GNKB                482    0.348  38.405  -1.127   0.386   0.097   0.517
E G  4GNKB                483    0.423  33.322  -0.805   0.386   0.097   0.517
E R  4GNKB                484    0.505 115.737  -0.731   0.386   0.097   0.517
E K  4GNKB                485    0.580 119.244  -1.183   0.386   0.097   0.517
E R  4GNKB                486    0.496 113.676  -1.171   0.278   0.093   0.628
E P  4GNKB                487    0.588  83.494  -1.396   0.278   0.093   0.628
E L  4GNKB                488    0.352  64.543  -1.375   0.278   0.093   0.628
E E  4GNKB                489    0.530  92.574  -1.561   0.278   0.093   0.628
E Q  4GNKB                490    0.516  92.193  -1.039   0.278   0.093   0.628
E S  4GNKB                491    0.488  57.240  -1.091   0.278   0.093   0.628
E N  4GNKB                492    0.466  68.266  -2.001   0.268   0.043   0.689
E S  4GNKB                493    0.608  71.234  -0.902   0.268   0.043   0.689
E A  4GNKB                494    0.432  47.562  -1.211   0.354   0.048   0.598
E L  4GNKB                495    0.502  91.935  -1.092   0.354   0.048   0.598
E S  4GNKB                496    0.525  61.565  -1.137   0.354   0.048   0.598
E E  4GNKB                497    0.480  83.786  -1.308   0.354   0.048   0.598
E S  4GNKB                498    0.627  73.520  -2.213   0.268   0.043   0.689
E S  4GNKB                499    0.528  61.858  -1.694   0.268   0.043   0.689
E A  4GNKB                500    0.426  46.978  -1.354   0.268   0.043   0.689
E A  4GNKB                501    0.529  58.307  -0.881   0.354   0.048   0.598
E T  4GNKB                502    0.555  76.992  -1.026   0.354   0.048   0.598
E E  4GNKB                503    0.499  87.140  -0.959   0.268   0.043   0.689
E P  4GNKB                504    0.606  85.949  -1.686   0.268   0.043   0.689
E S  4GNKB                505    0.437  51.263  -0.760   0.268   0.043   0.689
E S  4GNKB                506    0.347  40.692  -0.943   0.354   0.048   0.598
E P  4GNKB                507    0.527  74.739  -1.137   0.354   0.048   0.598
E Q  4GNKB                508    0.592 105.678  -1.381   0.354   0.048   0.598
E L  4GNKB                509    0.365  66.813  -1.139   0.268   0.043   0.689
E G  4GNKB                510    0.600  47.236  -2.423   0.184   0.043   0.773
E S  4GNKB                511    0.542  63.569  -0.700   0.113   0.043   0.844
E P  4GNKB                512    0.492  69.772  -1.380   0.184   0.043   0.773
E S  4GNKB                513    0.510  59.807  -1.628   0.181   0.016   0.803
E S  4GNKB                514    0.515  60.335  -1.360   0.339   0.016   0.645
E D  4GNKB                515    0.583  84.054  -1.754   0.430   0.016   0.555
E S  4GNKB                516    0.517  60.581  -1.083   0.430   0.016   0.555
E C  4GNKB                517    0.304  42.625  -1.004   0.430   0.016   0.555
E P  4GNKB                518    0.470  66.636  -0.526   0.430   0.016   0.555
E G  4GNKB                519    0.517  40.649  -0.700   0.430   0.016   0.555
E L  4GNKB                520    0.307  56.212  -0.982   0.455   0.046   0.498
E S  4GNKB                521    0.472  55.272  -0.705   0.354   0.048   0.598
E N  4GNKB                522    0.559  81.764  -0.778   0.354   0.048   0.598
E G  4GNKB                523    0.518  40.743  -0.998   0.278   0.093   0.628
E E  4GNKB                524    0.504  88.136  -0.875   0.278   0.093   0.628
E E  4GNKB                525    0.445  77.724  -0.539   0.278   0.093   0.628
E V  4GNKB                526    0.364  55.993  -0.801   0.278   0.093   0.628
E G  4GNKB                527    0.488  38.398  -1.028   0.191   0.086   0.723
E L  4GNKB                528    0.494  90.451  -1.418   0.191   0.086   0.723
E E  4GNKB                529    0.536  93.674  -1.690   0.113   0.043   0.844
E K  4GNKB                530    0.486  99.929  -1.396   0.113   0.043   0.844
E P  4GNKB                531    0.403  57.228  -1.835   0.053   0.043   0.903
E S  4GNKB                532    0.464  54.381  -1.528   0.113   0.043   0.844
E L  4GNKB                533    0.329  60.167  -1.257   0.113   0.043   0.844
E E  4GNKB                534    0.591 103.195  -0.808   0.181   0.016   0.803
E P  4GNKB                535    0.329  46.614  -1.656   0.257   0.016   0.727
E Q  4GNKB                536    0.413  73.744  -1.099   0.430   0.016   0.555
E K  4GNKB                537    0.556 114.451  -0.777   0.622   0.015   0.363
E S  4GNKB                538    0.521  61.014  -1.219   0.622   0.015   0.363
E L  4GNKB                539    0.312  57.127  -1.688   0.522   0.016   0.462
E G  4GNKB                540    0.345  27.159  -1.705   0.522   0.016   0.462
E D  4GNKB                541    0.532  76.690  -1.249   0.455   0.046   0.498
E E  4GNKB                542    0.503  87.927  -1.240   0.455   0.046   0.498
E G  4GNKB                543    0.348  27.403  -1.546   0.455   0.046   0.498
E L  4GNKB                544    0.324  59.379  -1.031   0.455   0.046   0.498
E N  4GNKB                545    0.477  69.833  -0.915   0.455   0.046   0.498
E R  4GNKB                546    0.583 133.415  -0.723   0.354   0.048   0.598
E G  4GNKB                547    0.461  36.289  -1.210   0.278   0.093   0.628
E P  4GNKB                548    0.423  59.981  -0.875   0.278   0.093   0.628
E Y  4GNKB                549    0.495 105.782  -0.631   0.199   0.152   0.649
E V  4GNKB                550    0.477  73.346  -1.340   0.199   0.152   0.649
E L  4GNKB                551    0.481  88.034  -1.072   0.191   0.086   0.723
E G  4GNKB                552    0.447  35.155  -1.323   0.113   0.043   0.844
E P  4GNKB                553    0.510  72.355  -1.275   0.113   0.043   0.844
E A  4GNKB                554    0.541  59.629  -1.161   0.184   0.043   0.773
E D  4GNKB                555    0.562  80.999  -0.554   0.268   0.043   0.689
E R  4GNKB                556    0.363  83.058  -0.421   0.354   0.048   0.598
E E  4GNKB                557    0.564  98.478  -0.901   0.354   0.048   0.598
E D  4GNKB                558    0.486  70.090  -1.198   0.561   0.047   0.393
E E  4GNKB                559    0.477  83.262  -1.087   0.561   0.047   0.393
E E  4GNKB                560    0.430  75.104  -1.219   0.561   0.047   0.393
E E  4GNKB                561    0.532  92.923  -0.900   0.561   0.047   0.393
E D  4GNKB                562    0.526  75.782  -0.895   0.455   0.046   0.498
E E  4GNKB                563    0.524  91.560  -1.236   0.455   0.046   0.498
E E  4GNKB                564    0.538  93.989  -1.500   0.354   0.048   0.598
E E  4GNKB                565    0.499  87.175  -0.825   0.455   0.046   0.498
E E  4GNKB                566    0.391  68.290  -1.046   0.522   0.016   0.462
E E  4GNKB                567    0.486  84.992  -0.812   0.522   0.016   0.462
E Q  4GNKB                568    0.495  88.353  -1.315   0.522   0.016   0.462
E T  4GNKB                569    0.503  69.711  -1.101   0.622   0.015   0.363
E D  4GNKB                570    0.597  85.956  -0.503   0.622   0.015   0.363
E P  4GNKB                571    0.372  52.829  -1.106   0.622   0.015   0.363
E K  4GNKB                572    0.458  94.128  -0.655   0.622   0.015   0.363
E K  4GNKB                573    0.615 126.485  -0.934   0.522   0.016   0.462
E P  4GNKB                574    0.575  81.564  -1.289   0.430   0.016   0.555
E T  4GNKB                575    0.473  65.619  -1.067   0.339   0.016   0.645
E T  4GNKB                576    0.394  54.634  -0.900   0.257   0.016   0.727
E D  4GNKB                577    0.623  89.803  -1.074   0.268   0.043   0.689
E E  4GNKB                578    0.592 103.335  -0.843   0.354   0.048   0.598
E G  4GNKB                579    0.442  34.793  -1.407   0.257   0.016   0.727
E T  4GNKB                580    0.389  53.940  -0.716   0.339   0.016   0.645
E A  4GNKB                581    0.549  60.456  -0.695   0.339   0.016   0.645
E S  4GNKB                582    0.554  64.917  -0.842   0.430   0.016   0.555
E S  4GNKB                583    0.348  40.774  -0.896   0.430   0.016   0.555
E E  4GNKB                584    0.387  67.609  -0.267   0.522   0.016   0.462
B V  4GNKB                585    0.227  34.859  -1.219   0.622   0.015   0.363
E N  4GNKB                586    0.529  77.446   0.215   0.751   0.050   0.199
B A  4GNKB                587    0.199  21.963  -0.251   0.717   0.014   0.269
B T  4GNKB                588    0.112  15.604  -0.695   0.717   0.014   0.269
E E  4GNKB                589    0.439  76.781   0.145   0.717   0.014   0.269
E E  4GNKB                590    0.423  73.968  -0.908   0.717   0.014   0.269
B M  4GNKB                591    0.105  20.910  -0.385   0.622   0.015   0.363
B S  4GNKB                592    0.207  24.307  -1.389   0.522   0.016   0.462
E T  4GNKB                593    0.427  59.267   0.183   0.354   0.048   0.598
B L  4GNKB                594    0.060  11.023  -0.391   0.307   0.165   0.527
B V  4GNKB                595    0.085  13.018  -0.042   0.252   0.423   0.325
E N  4GNKB                596    0.354  51.752  -0.775   0.159   0.516   0.325
B Y  4GNKB                597    0.178  38.103   0.400   0.148   0.418   0.435
B I  4GNKB                598    0.068  12.654  -1.874   0.066   0.296   0.638
E E  4GNKB                599    0.310  54.209  -0.220   0.021   0.279   0.699
E P  4GNKB                600    0.334  47.466  -1.787   0.019   0.141   0.840
B V  4GNKB                601    0.212  32.631  -0.493   0.004   0.138   0.858
E K  4GNKB                602    0.454  93.429  -0.367   0.018   0.088   0.893
B F  4GNKB                603    0.185  37.049  -1.812   0.018   0.047   0.935
E K  4GNKB                604    0.580 119.285  -0.827   0.018   0.019   0.964
E S  4GNKB                605    0.326  38.219  -2.068   0.018   0.047   0.935
B F  4GNKB                606    0.113  22.639  -0.639   0.052   0.084   0.864
E E  4GNKB                607    0.503  87.804  -0.421   0.113   0.043   0.844
E A  4GNKB                608    0.488  53.767  -0.020   0.184   0.043   0.773
E A  4GNKB                609    0.406  44.686  -1.723   0.184   0.043   0.773
E R  4GNKB                610    0.567 129.774  -0.778   0.184   0.043   0.773
E K  4GNKB                611    0.512 105.421  -1.026   0.113   0.043   0.844
E R  4GNKB                612    0.485 110.996  -0.965   0.053   0.043   0.903
E N  4GNKB                613    0.370  54.139  -1.297   0.018   0.047   0.935
E K  4GNKB                614    0.290  59.632  -0.111   0.005   0.045   0.951
B C  4GNKB                615    0.096  13.464  -0.377   0.005   0.262   0.733
B F  4GNKB                616    0.082  16.357   0.022   0.003   0.718   0.279
B E  4GNKB                617    0.070  12.246  -0.231   0.001   0.900   0.099
B M  4GNKB                618    0.041   8.224   0.074   0.001   0.900   0.099
B S  4GNKB                619    0.033   3.844  -0.348   0.001   0.900   0.099
B S  4GNKB                620    0.032   3.727  -1.816   0.003   0.718   0.279
B F  4GNKB                621    0.099  19.829  -0.102   0.005   0.336   0.660
E V  4GNKB                622    0.284  43.589  -0.107   0.018   0.088   0.893
B E  4GNKB                623    0.171  29.909  -1.358   0.600   0.003   0.397
E T  4GNKB                624    0.533  73.927   0.106   0.694   0.003   0.303
E K  4GNKB                625    0.289  59.488   0.703   0.694   0.003   0.303
B A  4GNKB                626    0.041   4.562  -1.170   0.923   0.002   0.076
E M  4GNKB                627    0.301  60.290   0.488   0.923   0.002   0.076
E E  4GNKB                628    0.522  91.193   1.295   0.970   0.001   0.030
B Q  4GNKB                629    0.108  19.200   0.313   0.970   0.001   0.030
B L  4GNKB                630    0.042   7.763  -1.224   0.923   0.002   0.076
E T  4GNKB                631    0.532  73.802   1.154   0.923   0.002   0.076
E K  4GNKB                632    0.526 108.280   0.747   0.858   0.002   0.139
B S  4GNKB                633    0.243  28.444   0.252   0.502   0.002   0.495
B P  4GNKB                634    0.262  37.192  -0.604   0.600   0.003   0.397
E M  4GNKB                635    0.503 100.610   0.338   0.694   0.003   0.303
E E  4GNKB                636    0.398  69.496   1.059   0.802   0.014   0.185
B F  4GNKB                637    0.034   6.764  -0.058   0.879   0.010   0.111
B V  4GNKB                638    0.156  23.946   0.160   0.879   0.010   0.111
E E  4GNKB                639    0.584 102.007   1.455   0.879   0.010   0.111
B Y  4GNKB                640    0.156  33.359   0.293   0.802   0.014   0.185
B N  4GNKB                641    0.069  10.131  -1.099   0.522   0.016   0.462
E K  4GNKB                642    0.406  83.494  -0.425   0.181   0.016   0.803
E Q  4GNKB                643    0.392  70.011   0.491   0.052   0.084   0.864
B Q  4GNKB                644    0.116  20.771   0.154   0.021   0.279   0.699
B L  4GNKB                645    0.035   6.390  -0.184   0.004   0.616   0.381
B S  4GNKB                646    0.034   4.043  -0.414   0.002   0.816   0.182
B R  4GNKB                647    0.065  14.931  -0.185   0.011   0.918   0.071
B I  4GNKB                648    0.045   8.399   0.060   0.018   0.846   0.136
B Y  4GNKB                649    0.123  26.178   1.210   0.004   0.420   0.576
B P  4GNKB                650    0.090  12.714  -0.513   0.004   0.197   0.799
E K  4GNKB                651    0.401  82.527  -0.221   0.018   0.088   0.893
B G  4GNKB                652    0.290  22.815  -1.981   0.019   0.141   0.840
B T  4GNKB                653    0.210  29.141   0.037   0.022   0.359   0.619
B R  4GNKB                654    0.209  47.953  -0.981   0.021   0.451   0.528
B V  4GNKB                655    0.269  41.345  -0.182   0.021   0.279   0.699
E D  4GNKB                656    0.513  73.938  -0.910   0.018   0.088   0.893
E S  4GNKB                657    0.318  37.270  -2.047   0.018   0.047   0.935
E S  4GNKB                658    0.309  36.238  -1.678   0.052   0.084   0.864
B N  4GNKB                659    0.258  37.742  -1.252   0.052   0.084   0.864
B Y  4GNKB                660    0.154  32.888  -1.155   0.053   0.043   0.903
B M  4GNKB                661    0.260  52.046  -0.215   0.053   0.043   0.903
B P  4GNKB                662    0.138  19.568  -1.711   0.184   0.043   0.773
B Q  4GNKB                663    0.170  30.380  -1.125   0.278   0.093   0.628
E L  4GNKB                664    0.308  56.322  -0.192   0.354   0.048   0.598
B F  4GNKB                665    0.085  17.140  -0.436   0.354   0.048   0.598
B W  4GNKB                666    0.091  21.789  -1.187   0.278   0.093   0.628
B N  4GNKB                667    0.210  30.685  -1.373   0.191   0.086   0.723
B V  4GNKB                668    0.099  15.232  -0.240   0.056   0.142   0.802
B G  4GNKB                669    0.039   3.085  -1.626   0.021   0.279   0.699
B C  4GNKB                670    0.022   3.145   0.617   0.021   0.451   0.528
B Q  4GNKB                671    0.049   8.751  -0.244   0.023   0.655   0.322
B L  4GNKB                672    0.032   5.786   0.573   0.021   0.756   0.223
B V  4GNKB                673    0.036   5.549  -0.053   0.021   0.756   0.223
B A  4GNKB                674    0.038   4.166  -1.564   0.022   0.552   0.426
B L  4GNKB                675    0.064  11.755  -0.189   0.020   0.205   0.775
B N  4GNKB                676    0.176  25.810  -1.028   0.052   0.084   0.864
B F  4GNKB                677    0.171  34.259  -0.446   0.113   0.087   0.800
B Q  4GNKB                678    0.215  38.381  -0.512   0.125   0.227   0.648
E T  4GNKB                679    0.261  36.159  -1.046   0.118   0.150   0.732
B L  4GNKB                680    0.218  39.934  -0.065   0.278   0.093   0.628
B D  4GNKB                681    0.201  29.036  -0.987   0.268   0.043   0.689
B V  4GNKB                682    0.236  36.212  -0.023   0.802   0.014   0.185
B A  4GNKB                683    0.088   9.731  -1.591   0.802   0.014   0.185
B M  4GNKB                684    0.052  10.325   0.204   0.879   0.010   0.111
B Q  4GNKB                685    0.107  19.021  -0.281   0.938   0.007   0.055
B L  4GNKB                686    0.047   8.679  -0.450   0.938   0.007   0.055
B N  4GNKB                687    0.029   4.246  -0.162   0.879   0.010   0.111
B A  4GNKB                688    0.114  12.530   0.277   0.879   0.010   0.111
B G  4GNKB                689    0.059   4.635  -1.929   0.879   0.010   0.111
B V  4GNKB                690    0.088  13.526   1.162   0.879   0.010   0.111
B F  4GNKB                691    0.032   6.402  -0.358   0.802   0.014   0.185
E E  4GNKB                692    0.359  62.787   0.518   0.751   0.050   0.199
E Y  4GNKB                693    0.492 105.226  -0.980   0.455   0.046   0.498
B N  4GNKB                694    0.306  44.740  -1.259   0.115   0.016   0.868
E G  4GNKB                695    0.443  34.840  -1.985   0.003   0.003   0.994
E R  4GNKB                696    0.385  88.234  -1.224   0.005   0.015   0.979
B S  4GNKB                697    0.115  13.431  -0.719   0.004   0.138   0.858
B G  4GNKB                698    0.061   4.801  -0.914   0.005   0.336   0.660
B Y  4GNKB                699    0.086  18.314   0.553   0.004   0.616   0.381
B L  4GNKB                700    0.043   7.928  -0.354   0.003   0.718   0.279
B L  4GNKB                701    0.102  18.768  -0.054   0.023   0.655   0.322
B K  4GNKB                702    0.152  31.349   0.850   0.019   0.141   0.840
B P  4GNKB                703    0.113  16.006  -0.112   0.058   0.017   0.925
E E  4GNKB                704    0.532  92.958  -0.057   0.184   0.043   0.773
B F  4GNKB                705    0.212  42.488  -1.259   0.199   0.152   0.649
B M  4GNKB                706    0.096  19.110  -1.675   0.125   0.227   0.648
B R  4GNKB                707    0.189  43.235  -0.455   0.118   0.150   0.732
E R  4GNKB                708    0.492 112.668  -0.216   0.052   0.084   0.864
E P  4GNKB                709    0.599  84.998  -1.996   0.058   0.017   0.925
E D  4GNKB                710    0.634  91.431  -2.055   0.018   0.019   0.964
E K  4GNKB                711    0.540 111.119  -1.396   0.018   0.019   0.964
E S  4GNKB                712    0.554  64.941  -1.139   0.018   0.047   0.935
B F  4GNKB                713    0.178  35.644  -1.535   0.018   0.047   0.935
E D  4GNKB                714    0.421  60.637  -0.978   0.018   0.019   0.964
E P  4GNKB                715    0.325  46.132  -1.833   0.058   0.017   0.925
E F  4GNKB                716    0.449  90.014  -1.489   0.053   0.043   0.903
E T  4GNKB                717    0.369  51.111  -0.564   0.053   0.043   0.903
E E  4GNKB                718    0.651 113.747  -0.340   0.113   0.043   0.844
E V  4GNKB                719    0.514  79.048  -0.668   0.113   0.087   0.800
E I  4GNKB                720    0.443  82.048  -1.036   0.052   0.084   0.864
B V  4GNKB                721    0.140  21.472  -0.747   0.052   0.084   0.864
E D  4GNKB                722    0.637  91.792  -0.020   0.018   0.047   0.935
E G  4GNKB                723    0.433  34.046  -1.223   0.018   0.088   0.893
B I  4GNKB                724    0.249  45.991  -0.023   0.005   0.262   0.733
E V  4GNKB                725    0.357  54.856  -0.013   0.004   0.420   0.576
E A  4GNKB                726    0.364  40.113   0.579   0.004   0.616   0.381
B N  4GNKB                727    0.140  20.467   0.060   0.001   0.900   0.099
E A  4GNKB                728    0.309  34.019   1.133   0.001   0.959   0.040
B L  4GNKB                729    0.038   6.903   0.292   0.001   0.959   0.040
E R  4GNKB                730    0.229  52.510   1.168   0.001   0.959   0.040
B V  4GNKB                731    0.026   3.935   0.853   0.001   0.959   0.040
E K  4GNKB                732    0.282  57.966   1.506   0.001   0.900   0.099
B V  4GNKB                733    0.027   4.150   0.403   0.021   0.756   0.223
B I  4GNKB                734    0.216  39.923   0.484   0.021   0.451   0.528
B S  4GNKB                735    0.178  20.850  -1.020   0.020   0.205   0.775
B G  4GNKB                736    0.124   9.790  -2.848   0.019   0.141   0.840
E Q  4GNKB                737    0.317  56.580  -0.488   0.019   0.141   0.840
B F  4GNKB                738    0.251  50.416  -1.774   0.019   0.141   0.840
B L  4GNKB                739    0.090  16.406  -0.327   0.018   0.047   0.935
E S  4GNKB                740    0.327  38.324  -1.362   0.018   0.088   0.893
E D  4GNKB                741    0.533  76.820  -1.170   0.018   0.047   0.935
E R  4GNKB                742    0.376  85.990  -0.963   0.005   0.015   0.979
E K  4GNKB                743    0.588 120.910  -1.543   0.003   0.003   0.994
B V  4GNKB                744    0.306  47.078  -2.311   0.003   0.003   0.994
E G  4GNKB                745    0.299  23.539  -1.267   0.005   0.015   0.979
B I  4GNKB                746    0.039   7.178  -0.854   0.004   0.138   0.858
B Y  4GNKB                747    0.138  29.597  -0.142   0.003   0.718   0.279
B V  4GNKB                748    0.019   2.859   1.513   0.001   0.900   0.099
B E  4GNKB                749    0.167  29.192   1.221   0.001   0.959   0.040
B V  4GNKB                750    0.018   2.767   1.263   0.001   0.959   0.040
B D  4GNKB                751    0.072  10.318   0.078   0.001   0.959   0.040
B M  4GNKB                752    0.026   5.123   0.265   0.001   0.959   0.040
B F  4GNKB                753    0.126  25.328   0.957   0.001   0.900   0.099
B G  4GNKB                754    0.101   7.933  -1.072   0.002   0.816   0.182
B L  4GNKB                755    0.112  20.562   0.675   0.004   0.420   0.576
E P  4GNKB                756    0.362  51.396  -0.274   0.004   0.197   0.799
E V  4GNKB                757    0.486  74.683  -1.004   0.019   0.141   0.840
E D  4GNKB                758    0.435  62.727  -1.023   0.018   0.047   0.935
E T  4GNKB                759    0.586  81.292  -1.538   0.018   0.047   0.935
E R  4GNKB                760    0.580 132.774  -0.230   0.018   0.088   0.893
E R  4GNKB                761    0.553 126.706   0.221   0.021   0.279   0.699
E K  4GNKB                762    0.502 103.364   0.373   0.004   0.514   0.481
E Y  4GNKB                763    0.389  83.044   0.060   0.003   0.718   0.279
E R  4GNKB                764    0.415  94.943   0.050   0.002   0.816   0.182
B T  4GNKB                765    0.262  36.353  -0.705   0.002   0.816   0.182
E R  4GNKB                766    0.442 101.332   0.595   0.002   0.816   0.182
B T  4GNKB                767    0.266  36.950   0.582   0.018   0.846   0.136
B S  4GNKB                768    0.122  14.298  -0.011   0.023   0.655   0.322
E Q  4GNKB                769    0.625 111.571   0.152   0.004   0.420   0.576
E G  4GNKB                770    0.643  50.573  -1.688   0.018   0.047   0.935
E N  4GNKB                771    0.440  64.357  -1.172   0.018   0.019   0.964
E S  4GNKB                772    0.366  42.872  -1.491   0.005   0.045   0.951
B F  4GNKB                773    0.252  50.596  -0.589   0.018   0.088   0.893
B N  4GNKB                774    0.246  35.941   0.055   0.004   0.138   0.858
B P  4GNKB                775    0.089  12.700  -0.748   0.004   0.197   0.799
E V  4GNKB                776    0.288  44.327   0.370   0.022   0.552   0.426
B W  4GNKB                777    0.113  27.128  -0.082   0.022   0.552   0.426
E D  4GNKB                778    0.464  66.934  -0.308   0.004   0.138   0.858
E E  4GNKB                779    0.578 101.012  -0.578   0.005   0.045   0.951
E E  4GNKB                780    0.618 107.947  -0.147   0.005   0.262   0.733
E P  4GNKB                781    0.358  50.758   0.638   0.004   0.514   0.481
B F  4GNKB                782    0.111  22.177  -0.600   0.022   0.552   0.426
E D  4GNKB                783    0.473  68.203   0.219   0.023   0.655   0.322
B F  4GNKB                784    0.150  30.185  -0.956   0.022   0.552   0.426
E P  4GNKB                785    0.340  48.289  -0.030   0.022   0.552   0.426
E K  4GNKB                786    0.473  97.317   0.326   0.023   0.655   0.322
B V  4GNKB                787    0.073  11.159   0.272   0.021   0.756   0.223
E V  4GNKB                788    0.346  53.211   0.631   0.004   0.616   0.381
B L  4GNKB                789    0.096  17.541   1.029   0.022   0.359   0.619
B P  4GNKB                790    0.233  33.105   0.529   0.053   0.043   0.903
E T  4GNKB                791    0.265  36.797  -0.490   0.052   0.084   0.864
B L  4GNKB                792    0.078  14.190   0.502   0.069   0.386   0.545
B A  4GNKB                793    0.021   2.281   0.656   0.023   0.655   0.322
B S  4GNKB                794    0.044   5.122  -0.066   0.011   0.918   0.071
B L  4GNKB                795    0.013   2.454   1.692   0.006   0.962   0.032
B R  4GNKB                796    0.077  17.587   0.915   0.006   0.962   0.032
B I  4GNKB                797    0.018   3.312   1.383   0.002   0.985   0.013
B A  4GNKB                798    0.065   7.152   0.991   0.002   0.985   0.013
B A  4GNKB                799    0.021   2.270   0.828   0.006   0.962   0.032
B F  4GNKB                800    0.219  43.873   1.276   0.006   0.962   0.032
B E  4GNKB                801    0.187  32.704  -0.678   0.002   0.816   0.182
E E  4GNKB                802    0.442  77.287  -0.135   0.004   0.197   0.799
E G  4GNKB                803    0.707  55.672  -0.751   0.005   0.015   0.979
E G  4GNKB                804    0.456  35.919  -1.409   0.005   0.015   0.979
E K  4GNKB                805    0.448  92.154  -0.073   0.022   0.359   0.619
B F  4GNKB                806    0.085  17.059   0.116   0.002   0.816   0.182
B V  4GNKB                807    0.045   6.963   0.112   0.011   0.918   0.071
B G  4GNKB                808    0.023   1.786   0.160   0.001   0.959   0.040
B H  4GNKB                809    0.065  11.860   0.262   0.001   0.959   0.040
B R  4GNKB                810    0.038   8.771   0.455   0.001   0.900   0.099
B I  4GNKB                811    0.030   5.624   0.088   0.001   0.900   0.099
B L  4GNKB                812    0.038   7.013   0.925   0.002   0.816   0.182
B P  4GNKB                813    0.156  22.122   0.187   0.004   0.616   0.381
B V  4GNKB                814    0.055   8.500  -0.729   0.020   0.205   0.775
E S  4GNKB                815    0.429  50.244  -0.639   0.113   0.043   0.844
E A  4GNKB                816    0.279  30.768  -1.040   0.053   0.043   0.903
B I  4GNKB                817    0.124  22.866  -1.388   0.113   0.087   0.800
E R  4GNKB                818    0.287  65.792   0.040   0.113   0.087   0.800
E S  4GNKB                819    0.311  36.391  -0.308   0.125   0.227   0.648
B G  4GNKB                820    0.051   4.037  -2.359   0.135   0.317   0.548
B Y  4GNKB                821    0.123  26.285   0.307   0.159   0.516   0.325
B H  4GNKB                822    0.226  41.073   0.371   0.265   0.667   0.069
B Y  4GNKB                823    0.147  31.371   1.077   0.387   0.545   0.068
B V  4GNKB                824    0.041   6.348   0.645   0.731   0.235   0.034
E C  4GNKB                825    0.356  49.982   0.725   0.870   0.077   0.053
B L  4GNKB                826    0.222  40.557  -0.983   0.831   0.044   0.125
B R  4GNKB                827    0.241  55.097  -0.094   0.751   0.050   0.199
E N  4GNKB                828    0.399  58.458  -0.039   0.522   0.016   0.462
E E  4GNKB                829    0.573 100.086  -1.258   0.339   0.016   0.645
E A  4GNKB                830    0.532  58.637  -0.919   0.181   0.016   0.803
E N  4GNKB                831    0.493  72.146  -1.513   0.058   0.017   0.925
E Q  4GNKB                832    0.528  94.247  -0.540   0.053   0.043   0.903
E P  4GNKB                833    0.403  57.200  -0.488   0.113   0.087   0.800
B L  4GNKB                834    0.118  21.642   0.205   0.113   0.087   0.800
E C  4GNKB                835    0.493  69.189  -1.146   0.184   0.043   0.773
E L  4GNKB                836    0.415  75.913  -0.893   0.522   0.016   0.462
B P  4GNKB                837    0.276  39.122  -1.859   0.622   0.015   0.363
E A  4GNKB                838    0.355  39.132  -0.856   0.802   0.014   0.185
B L  4GNKB                839    0.239  43.834  -0.494   0.802   0.014   0.185
B L  4GNKB                840    0.185  33.855  -1.028   0.802   0.014   0.185
B I  4GNKB                841    0.218  40.367  -0.202   0.923   0.002   0.076
E Y  4GNKB                842    0.387  82.766  -0.234   0.970   0.001   0.030
B T  4GNKB                843    0.216  29.904  -1.100   0.970   0.001   0.030
B E  4GNKB                844    0.258  45.055  -0.388   0.938   0.007   0.055
E A  4GNKB                845    0.346  38.096  -1.284   0.858   0.002   0.139
E S  4GNKB                846    0.397  46.517  -0.827   0.694   0.003   0.303
E D  4GNKB                847    0.319  45.939  -1.691   0.502   0.002   0.495
E Y  4GNKB                848    0.392  83.813  -1.111   0.430   0.016   0.555
E I  4GNKB                849    0.368  68.006  -1.330   0.339   0.016   0.645
E P  4GNKB                850    0.391  55.426  -1.404   0.257   0.016   0.727
E D  4GNKB                851    0.411  59.196  -1.436   0.257   0.016   0.727
E D  4GNKB                852    0.435  62.626  -1.663   0.257   0.016   0.727
E H  4GNKB                853    0.334  60.809  -0.956   0.522   0.016   0.462
E Q  4GNKB                854    0.505  90.247  -1.214   0.600   0.003   0.397
E D  4GNKB                855    0.516  74.370  -1.278   0.600   0.003   0.397
E Y  4GNKB                856    0.303  64.751  -0.611   0.694   0.003   0.303
E A  4GNKB                857    0.412  45.402  -0.573   0.694   0.003   0.303
E E  4GNKB                858    0.607 105.990  -0.143   0.782   0.003   0.216
E A  4GNKB                859    0.369  40.642  -0.587   0.694   0.003   0.303
E L  4GNKB                860    0.371  67.875  -1.007   0.694   0.003   0.303
E I  4GNKB                861    0.547 101.250  -0.535   0.782   0.003   0.216
E N  4GNKB                862    0.584  85.483   0.351   0.782   0.003   0.216
E P  4GNKB                863    0.413  58.591  -0.441   0.858   0.002   0.139
E I  4GNKB                864    0.467  86.432  -0.099   0.923   0.002   0.076
E K  4GNKB                865    0.586 120.499   0.043   0.923   0.002   0.076
E H  4GNKB                866    0.447  81.346  -0.146   0.858   0.002   0.139
E V  4GNKB                867    0.342  52.565  -0.630   0.858   0.002   0.139
E S  4GNKB                868    0.548  64.261  -0.579   0.694   0.003   0.303
E L  4GNKB                869    0.462  84.556  -0.415   0.694   0.003   0.303
E M  4GNKB                870    0.396  79.260  -0.455   0.782   0.003   0.216
E D  4GNKB                871    0.463  66.689  -0.765   0.782   0.003   0.216
E Q  4GNKB                872    0.517  92.426  -0.284   0.782   0.003   0.216
E R  4GNKB                873    0.403  92.264  -0.100   0.923   0.002   0.076
E A  4GNKB                874    0.408  44.951  -0.517   0.923   0.002   0.076
E R  4GNKB                875    0.588 134.767   0.178   0.923   0.002   0.076
E Q  4GNKB                876    0.443  79.084   0.119   0.970   0.001   0.030
B L  4GNKB                877    0.246  45.079  -0.233   0.970   0.001   0.030
E A  4GNKB                878    0.608  67.035   0.306   0.970   0.001   0.030
E A  4GNKB                879    0.549  60.544   0.561   0.970   0.001   0.030
E L  4GNKB                880    0.320  58.519   0.321   0.970   0.001   0.030
E I  4GNKB                881    0.464  85.803   0.071   0.923   0.002   0.076
E G  4GNKB                882    0.618  48.629   0.066   0.923   0.002   0.076
E E  4GNKB                883    0.511  89.237  -0.090   0.923   0.002   0.076
E S  4GNKB                884    0.402  47.056  -0.248   0.923   0.002   0.076
E E  4GNKB                885    0.528  92.277  -0.027   0.923   0.002   0.076
E A  4GNKB                886    0.534  58.847   0.370   0.970   0.001   0.030
E Q  4GNKB                887    0.492  87.800   0.376   0.970   0.001   0.030
E A  4GNKB                888    0.497  54.736  -0.393   0.923   0.002   0.076
E G  4GNKB                889    0.530  41.727  -0.443   0.923   0.002   0.076
E Q  4GNKB                890    0.529  94.497  -0.109   0.923   0.002   0.076
E E  4GNKB                891    0.513  89.604  -0.078   0.923   0.002   0.076
E T  4GNKB                892    0.456  63.206  -0.363   0.923   0.002   0.076
E C  4GNKB                893    0.559  78.526  -0.143   0.858   0.002   0.139
E Q  4GNKB                894    0.515  91.943  -0.267   0.858   0.002   0.139
E D  4GNKB                895    0.326  47.020  -0.341   0.858   0.002   0.139
E T  4GNKB                896    0.612  84.926  -0.020   0.923   0.002   0.076
E Q  4GNKB                897    0.504  90.086   0.122   0.970   0.001   0.030
E S  4GNKB                898    0.471  55.166  -0.092   0.970   0.001   0.030
E Q  4GNKB                899    0.466  83.299  -0.049   0.970   0.001   0.030
E Q  4GNKB                900    0.552  98.569   0.138   0.970   0.001   0.030
E L  4GNKB                901    0.384  70.255  -0.126   0.923   0.002   0.076
E G  4GNKB                902    0.398  31.307  -1.030   0.858   0.002   0.139
E S  4GNKB                903    0.484  56.690  -0.598   0.858   0.002   0.139
E Q  4GNKB                904    0.496  88.532  -0.272   0.858   0.002   0.139
E P  4GNKB                905    0.415  58.945  -1.056   0.782   0.003   0.216
E S  4GNKB                906    0.478  56.033  -0.815   0.782   0.003   0.216
E S  4GNKB                907    0.600  70.261  -0.627   0.782   0.003   0.216
E N  4GNKB                908    0.467  68.413  -0.370   0.782   0.003   0.216
E P  4GNKB                909    0.485  68.779  -0.675   0.782   0.003   0.216
E T  4GNKB                910    0.483  66.950  -0.599   0.782   0.003   0.216
E P  4GNKB                911    0.581  82.387  -0.314   0.858   0.002   0.139
E S  4GNKB                912    0.522  61.167  -0.447   0.858   0.002   0.139
E P  4GNKB                913    0.499  70.851  -0.275   0.858   0.002   0.139
E L  4GNKB                914    0.410  75.126  -0.783   0.858   0.002   0.139
E D  4GNKB                915    0.561  80.797  -0.233   0.923   0.002   0.076
E A  4GNKB                916    0.521  57.403  -0.551   0.923   0.002   0.076
E S  4GNKB                917    0.466  54.662  -0.220   0.923   0.002   0.076
E P  4GNKB                918    0.469  66.594  -0.348   0.923   0.002   0.076
E R  4GNKB                919    0.532 121.828   0.191   0.923   0.002   0.076
E R  4GNKB                920    0.445 101.791  -0.049   0.858   0.002   0.139
E P  4GNKB                921    0.505  71.659  -0.139   0.858   0.002   0.139
E P  4GNKB                922    0.461  65.473  -0.738   0.858   0.002   0.139
E G  4GNKB                923    0.562  44.245  -0.714   0.858   0.002   0.139
E P  4GNKB                924    0.375  53.212  -0.474   0.923   0.002   0.076
E T  4GNKB                925    0.517  71.736  -0.281   0.923   0.002   0.076
E T  4GNKB                926    0.479  66.382  -0.481   0.923   0.002   0.076
E S  4GNKB                927    0.490  57.475  -0.353   0.923   0.002   0.076
E P  4GNKB                928    0.511  72.553  -0.358   0.923   0.002   0.076
E A  4GNKB                929    0.528  58.142  -0.183   0.923   0.002   0.076
E S  4GNKB                930    0.531  62.198  -0.135   0.923   0.002   0.076
E T  4GNKB                931    0.498  69.142  -0.121   0.923   0.002   0.076
E S  4GNKB                932    0.452  52.974  -0.287   0.923   0.002   0.076
E L  4GNKB                933    0.511  93.509  -0.556   0.858   0.002   0.139
E S  4GNKB                934    0.560  65.585  -0.117   0.858   0.002   0.139
E S  4GNKB                935    0.497  58.272   0.061   0.923   0.002   0.076
E P  4GNKB                936    0.495  70.269  -0.365   0.923   0.002   0.076
E G  4GNKB                937    0.590  46.449  -0.276   0.923   0.002   0.076
E Q  4GNKB                938    0.486  86.728  -0.268   0.858   0.002   0.139
E R  4GNKB                939    0.321  73.509  -0.470   0.858   0.002   0.139
E D  4GNKB                940    0.470  67.770  -0.752   0.923   0.002   0.076
E D  4GNKB                941    0.535  77.021  -0.148   0.923   0.002   0.076
E L  4GNKB                942    0.331  60.679  -0.121   0.970   0.001   0.030
E I  4GNKB                943    0.325  60.180  -0.357   0.970   0.001   0.030
E A  4GNKB                944    0.490  53.976  -0.011   0.970   0.001   0.030
E S  4GNKB                945    0.529  61.975   0.380   0.970   0.001   0.030
E I  4GNKB                946    0.383  70.929   0.175   0.970   0.001   0.030
E L  4GNKB                947    0.417  76.389   0.129   0.970   0.001   0.030
E S  4GNKB                948    0.523  61.296   0.238   0.970   0.001   0.030
E E  4GNKB                949    0.492  86.022   0.369   0.970   0.001   0.030
E V  4GNKB                950    0.419  64.370  -0.063   0.923   0.002   0.076
E A  4GNKB                951    0.419  46.152   0.111   0.858   0.002   0.139
E P  4GNKB                952    0.445  63.145  -0.168   0.782   0.003   0.216
E T  4GNKB                953    0.451  62.554  -0.119   0.858   0.002   0.139
E P  4GNKB                954    0.423  59.953  -0.068   0.858   0.002   0.139
E L  4GNKB                955    0.469  85.929   0.132   0.923   0.002   0.076
E D  4GNKB                956    0.572  82.497   0.135   0.923   0.002   0.076
E E  4GNKB                957    0.522  91.176   0.464   0.970   0.001   0.030
E L  4GNKB                958    0.354  64.854   0.147   0.970   0.001   0.030
E R  4GNKB                959    0.547 125.286   0.653   0.970   0.001   0.030
E G  4GNKB                960    0.472  37.154   0.766   0.970   0.001   0.030
E H  4GNKB                961    0.408  74.161   0.616   0.970   0.001   0.030
E K  4GNKB                962    0.494 101.698   0.776   0.970   0.001   0.030
E A  4GNKB                963    0.466  51.397   0.598   0.970   0.001   0.030
E L  4GNKB                964    0.342  62.639   0.802   0.970   0.001   0.030
E V  4GNKB                965    0.447  68.704   0.739   0.970   0.001   0.030
E K  4GNKB                966    0.597 122.844   1.068   0.970   0.001   0.030
E L  4GNKB                967    0.394  72.233   0.775   0.970   0.001   0.030
E R  4GNKB                968    0.287  65.769   0.871   0.970   0.001   0.030
E S  4GNKB                969    0.510  59.795   1.125   0.970   0.001   0.030
E R  4GNKB                970    0.538 123.110   1.325   0.970   0.001   0.030
E Q  4GNKB                971    0.302  53.919   1.091   0.970   0.001   0.030
E E  4GNKB                972    0.350  61.145   0.991   0.970   0.001   0.030
E R  4GNKB                973    0.565 129.477   1.418   0.970   0.001   0.030
E D  4GNKB                974    0.459  66.084   0.780   0.970   0.001   0.030
E L  4GNKB                975    0.303  55.553   0.814   0.970   0.001   0.030
E R  4GNKB                976    0.528 120.935   0.759   0.970   0.001   0.030
E E  4GNKB                977    0.526  91.910   1.190   0.970   0.001   0.030
E L  4GNKB                978    0.261  47.698   1.078   0.970   0.001   0.030
E R  4GNKB                979    0.437 100.096   0.849   0.970   0.001   0.030
E K  4GNKB                980    0.553 113.711   1.198   0.970   0.001   0.030
E K  4GNKB                981    0.482  99.209   1.191   0.970   0.001   0.030
E H  4GNKB                982    0.356  64.665   0.798   0.970   0.001   0.030
E Q  4GNKB                983    0.372  66.511   0.950   0.970   0.001   0.030
E R  4GNKB                984    0.590 135.064   1.622   0.970   0.001   0.030
E K  4GNKB                985    0.393  80.799   1.229   0.970   0.001   0.030
E A  4GNKB                986    0.321  35.374   1.273   0.970   0.001   0.030
E V  4GNKB                987    0.476  73.084   1.066   0.970   0.001   0.030
E T  4GNKB                988    0.528  73.220   1.412   0.970   0.001   0.030
B L  4GNKB                989    0.242  44.365   1.093   0.970   0.001   0.030
E T  4GNKB                990    0.385  53.372   1.145   0.970   0.001   0.030
E R  4GNKB                991    0.581 133.118   1.456   0.970   0.001   0.030
E R  4GNKB                992    0.467 106.989   1.439   0.970   0.001   0.030
B L  4GNKB                993    0.267  48.906   0.939   0.970   0.001   0.030
E L  4GNKB                994    0.443  81.205   0.739   0.970   0.001   0.030
E D  4GNKB                995    0.593  85.495   1.198   0.970   0.001   0.030
E G  4GNKB                996    0.415  32.684   0.996   0.970   0.001   0.030
E L  4GNKB                997    0.322  59.013   0.849   0.970   0.001   0.030
E A  4GNKB                998    0.525  57.855   0.659   0.970   0.001   0.030
E Q  4GNKB                999    0.542  96.855   0.957   0.970   0.001   0.030
E A  4GNKB               1000    0.360  39.617   0.397   0.923   0.002   0.076
E Q  4GNKB               1001    0.426  76.048   0.447   0.923   0.002   0.076
E A  4GNKB               1002    0.526  57.921   0.587   0.970   0.001   0.030
E E  4GNKB               1003    0.488  85.289   0.284   0.970   0.001   0.030
E G  4GNKB               1004    0.415  32.676   0.228   0.970   0.001   0.030
E R  4GNKB               1005    0.551 126.087   0.759   0.970   0.001   0.030
E C  4GNKB               1006    0.464  65.103   0.389   0.970   0.001   0.030
E R  4GNKB               1007    0.395  90.501   0.668   0.970   0.001   0.030
E L  4GNKB               1008    0.463  84.720   0.620   0.970   0.001   0.030
E R  4GNKB               1009    0.488 111.660   1.001   0.970   0.001   0.030
E P  4GNKB               1010    0.450  63.898   0.839   0.970   0.001   0.030
E G  4GNKB               1011    0.392  30.866   0.777   0.970   0.001   0.030
E A  4GNKB               1012    0.528  58.175   0.677   0.970   0.001   0.030
E L  4GNKB               1013    0.454  83.054   0.691   0.970   0.001   0.030
E G  4GNKB               1014    0.355  27.938   0.353   0.970   0.001   0.030
E G  4GNKB               1015    0.505  39.767   0.517   0.970   0.001   0.030
E A  4GNKB               1016    0.551  60.676   0.727   0.970   0.001   0.030
E A  4GNKB               1017    0.360  39.639   0.942   0.970   0.001   0.030
E D  4GNKB               1018    0.419  60.436   0.443   0.970   0.001   0.030
E V  4GNKB               1019    0.527  80.923   0.678   0.923   0.002   0.076
E E  4GNKB               1020    0.521  91.089   0.494   0.923   0.002   0.076
E D  4GNKB               1021    0.339  48.807   0.740   0.970   0.001   0.030
E T  4GNKB               1022    0.501  69.558   0.432   0.970   0.001   0.030
E K  4GNKB               1023    0.559 115.069   0.980   0.970   0.001   0.030
E E  4GNKB               1024    0.388  67.766   0.487   0.970   0.001   0.030
E G  4GNKB               1025    0.487  38.351   0.809   0.970   0.001   0.030
E E  4GNKB               1026    0.564  98.513   0.678   0.970   0.001   0.030
E D  4GNKB               1027    0.536  77.238   1.123   0.970   0.001   0.030
E E  4GNKB               1028    0.352  61.459   0.828   0.970   0.001   0.030
E A  4GNKB               1029    0.501  55.177   1.361   0.970   0.001   0.030
E K  4GNKB               1030    0.597 122.721   1.306   0.970   0.001   0.030
E R  4GNKB               1031    0.500 114.454   1.123   0.970   0.001   0.030
B Y  4GNKB               1032    0.253  54.045   1.041   0.970   0.001   0.030
E Q  4GNKB               1033    0.582 103.963   1.153   0.970   0.001   0.030
E E  4GNKB               1034    0.613 107.161   1.471   0.970   0.001   0.030
E F  4GNKB               1035    0.290  58.163   0.707   0.970   0.001   0.030
E Q  4GNKB               1036    0.458  81.763   0.583   0.970   0.001   0.030
E N  4GNKB               1037    0.622  91.119   0.999   0.970   0.001   0.030
E R  4GNKB               1038    0.598 137.056   1.543   0.970   0.001   0.030
E Q  4GNKB               1039    0.309  55.187   1.276   0.970   0.001   0.030
E V  4GNKB               1040    0.526  80.846   1.071   0.923   0.002   0.076
E Q  4GNKB               1041    0.581 103.731   1.402   0.923   0.002   0.076
E S  4GNKB               1042    0.467  54.686   1.311   0.970   0.001   0.030
E L  4GNKB               1043    0.288  52.714   1.028   0.970   0.001   0.030
E L  4GNKB               1044    0.521  95.395   0.895   0.970   0.001   0.030
E E  4GNKB               1045    0.561  98.059   1.235   0.970   0.001   0.030
E L  4GNKB               1046    0.266  48.668   0.988   0.970   0.001   0.030
E R  4GNKB               1047    0.395  90.524   0.660   0.970   0.001   0.030
E E  4GNKB               1048    0.593 103.545   1.115   0.970   0.001   0.030
E A  4GNKB               1049    0.506  55.717   1.212   0.970   0.001   0.030
E Q  4GNKB               1050    0.318  56.866   1.132   0.970   0.001   0.030
E V  4GNKB               1051    0.469  72.101   1.204   0.970   0.001   0.030
E D  4GNKB               1052    0.552  79.572   1.381   0.970   0.001   0.030
E A  4GNKB               1053    0.406  44.785   1.126   0.970   0.001   0.030
E E  4GNKB               1054    0.380  66.316   0.893   0.970   0.001   0.030
E A  4GNKB               1055    0.462  50.912   1.193   0.970   0.001   0.030
E Q  4GNKB               1056    0.517  92.336   1.304   0.970   0.001   0.030
E R  4GNKB               1057    0.398  91.096   1.432   0.970   0.001   0.030
E R  4GNKB               1058    0.391  89.585   0.799   0.970   0.001   0.030
E L  4GNKB               1059    0.448  81.956   1.097   0.970   0.001   0.030
E E  4GNKB               1060    0.550  96.068   1.099   0.970   0.001   0.030
E H  4GNKB               1061    0.412  74.852   1.100   0.970   0.001   0.030
E L  4GNKB               1062    0.328  60.057   0.970   0.970   0.001   0.030
E R  4GNKB               1063    0.588 134.560   1.610   0.970   0.001   0.030
E Q  4GNKB               1064    0.511  91.247   1.448   0.970   0.001   0.030
E A  4GNKB               1065    0.343  37.832   1.170   0.970   0.001   0.030
E L  4GNKB               1066    0.385  70.457   1.390   0.970   0.001   0.030
E Q  4GNKB               1067    0.577 103.106   1.455   0.970   0.001   0.030
E R  4GNKB               1068    0.427  97.852   1.614   0.970   0.001   0.030
E L  4GNKB               1069    0.294  53.776   0.978   0.970   0.001   0.030
E R  4GNKB               1070    0.484 110.722   1.390   0.970   0.001   0.030
E E  4GNKB               1071    0.524  91.543   1.383   0.970   0.001   0.030
E V  4GNKB               1072    0.391  60.051   1.083   0.970   0.001   0.030
E V  4GNKB               1073    0.325  50.029   0.580   0.970   0.001   0.030
E L  4GNKB               1074    0.537  98.361   0.694   0.970   0.001   0.030
E D  4GNKB               1075    0.595  85.740   1.098   0.970   0.001   0.030
E A  4GNKB               1076    0.474  52.235   1.060   0.970   0.001   0.030
E N  4GNKB               1077    0.360  52.675   0.816   0.923   0.002   0.076
E T  4GNKB               1078    0.543  75.383   1.109   0.970   0.001   0.030
E T  4GNKB               1079    0.592  82.041   1.499   0.970   0.001   0.030
E Q  4GNKB               1080    0.395  70.618   1.027   0.970   0.001   0.030
E F  4GNKB               1081    0.289  57.942   0.932   0.970   0.001   0.030
E K  4GNKB               1082    0.599 123.255   1.274   0.970   0.001   0.030
E R  4GNKB               1083    0.513 117.431   1.517   0.970   0.001   0.030
E L  4GNKB               1084    0.282  51.634   0.918   0.970   0.001   0.030
E K  4GNKB               1085    0.432  88.904   0.822   0.970   0.001   0.030
E E  4GNKB               1086    0.551  96.190   0.902   0.970   0.001   0.030
E M  4GNKB               1087    0.506 101.171   1.258   0.970   0.001   0.030
E N  4GNKB               1088    0.290  42.471   0.979   0.970   0.001   0.030
E E  4GNKB               1089    0.527  92.119   1.111   0.970   0.001   0.030
E R  4GNKB               1090    0.560 128.332   1.489   0.970   0.001   0.030
E E  4GNKB               1091    0.362  63.224   0.866   0.970   0.001   0.030
E K  4GNKB               1092    0.373  76.829   0.772   0.970   0.001   0.030
E K  4GNKB               1093    0.580 119.388   1.228   0.970   0.001   0.030
E E  4GNKB               1094    0.524  91.578   1.495   0.970   0.001   0.030
B L  4GNKB               1095    0.258  47.167   1.001   0.970   0.001   0.030
E Q  4GNKB               1096    0.431  76.959   1.156   0.970   0.001   0.030
E K  4GNKB               1097    0.572 117.722   1.654   0.970   0.001   0.030
E I  4GNKB               1098    0.477  88.171   1.393   0.970   0.001   0.030
E L  4GNKB               1099    0.303  55.443   1.171   0.970   0.001   0.030
E D  4GNKB               1100    0.517  74.529   0.819   0.970   0.001   0.030
E R  4GNKB               1101    0.559 127.942   1.447   0.970   0.001   0.030
E K  4GNKB               1102    0.482  99.250   1.168   0.970   0.001   0.030
E R  4GNKB               1103    0.442 101.104   0.788   0.970   0.001   0.030
E H  4GNKB               1104    0.489  88.913   1.147   0.970   0.001   0.030
E N  4GNKB               1105    0.567  82.950   1.181   0.970   0.001   0.030
E S  4GNKB               1106    0.399  46.751   1.143   0.970   0.001   0.030
E I  4GNKB               1107    0.394  72.946   0.843   0.970   0.001   0.030
E S  4GNKB               1108    0.531  62.280   0.974   0.970   0.001   0.030
E E  4GNKB               1109    0.538  94.024   1.226   0.970   0.001   0.030
E A  4GNKB               1110    0.405  44.642   1.077   0.970   0.001   0.030
E K  4GNKB               1111    0.468  96.268   0.847   0.970   0.001   0.030
E M  4GNKB               1112    0.501 100.170   0.840   0.970   0.001   0.030
E R  4GNKB               1113    0.449 102.890   0.911   0.970   0.001   0.030
E D  4GNKB               1114    0.523  75.364   0.863   0.970   0.001   0.030
E K  4GNKB               1115    0.453  93.244   1.073   0.970   0.001   0.030
E H  4GNKB               1116    0.462  84.001   0.778   0.970   0.001   0.030
E K  4GNKB               1117    0.480  98.715   1.102   0.970   0.001   0.030
E K  4GNKB               1118    0.460  94.663   1.012   0.970   0.001   0.030
E E  4GNKB               1119    0.473  82.686   0.879   0.970   0.001   0.030
E A  4GNKB               1120    0.443  48.863   1.024   0.970   0.001   0.030
E E  4GNKB               1121    0.442  77.182   0.982   0.970   0.001   0.030
E L  4GNKB               1122    0.403  73.734   0.954   0.970   0.001   0.030
E T  4GNKB               1123    0.483  66.937   0.984   0.970   0.001   0.030
E E  4GNKB               1124    0.489  85.498   0.705   0.970   0.001   0.030
E I  4GNKB               1125    0.373  68.987   0.846   0.970   0.001   0.030
E N  4GNKB               1126    0.472  69.086   0.495   0.970   0.001   0.030
E R  4GNKB               1127    0.495 113.355   0.960   0.970   0.001   0.030
E R  4GNKB               1128    0.486 111.363   1.099   0.970   0.001   0.030
E H  4GNKB               1129    0.462  83.965   0.897   0.970   0.001   0.030
E I  4GNKB               1130    0.346  64.065   1.014   0.970   0.001   0.030
E T  4GNKB               1131    0.516  71.555   0.966   0.970   0.001   0.030
E E  4GNKB               1132    0.500  87.280   0.843   0.970   0.001   0.030
E S  4GNKB               1133    0.392  45.907   0.692   0.970   0.001   0.030
E V  4GNKB               1134    0.464  71.394   0.636   0.970   0.001   0.030
E N  4GNKB               1135    0.587  85.966   1.026   0.970   0.001   0.030
E S  4GNKB               1136    0.429  50.291   0.703   0.970   0.001   0.030
E I  4GNKB               1137    0.312  57.701   1.143   0.970   0.001   0.030
E R  4GNKB               1138    0.527 120.729   1.117   0.970   0.001   0.030
E R  4GNKB               1139    0.510 116.813   1.507   0.970   0.001   0.030
B L  4GNKB               1140    0.233  42.644   0.620   0.970   0.001   0.030
E E  4GNKB               1141    0.424  74.038   0.804   0.970   0.001   0.030
E E  4GNKB               1142    0.531  92.696   1.139   0.970   0.001   0.030
E A  4GNKB               1143    0.507  55.816   1.419   0.970   0.001   0.030
E Q  4GNKB               1144    0.295  52.705   1.197   0.970   0.001   0.030
E K  4GNKB               1145    0.518 106.470   1.037   0.970   0.001   0.030
E Q  4GNKB               1146    0.574 102.445   1.516   0.970   0.001   0.030
E R  4GNKB               1147    0.442 101.287   0.914   0.970   0.001   0.030
E H  4GNKB               1148    0.325  59.045   0.960   0.970   0.001   0.030
E D  4GNKB               1149    0.571  82.223   1.048   0.970   0.001   0.030
E R  4GNKB               1150    0.566 129.706   1.572   0.970   0.001   0.030
E L  4GNKB               1151    0.290  53.007   1.009   0.970   0.001   0.030
E V  4GNKB               1152    0.481  73.884   0.942   0.970   0.001   0.030
E A  4GNKB               1153    0.557  61.348   1.191   0.970   0.001   0.030
E G  4GNKB               1154    0.510  40.161   1.227   0.970   0.001   0.030
E Q  4GNKB               1155    0.310  55.437   0.962   0.970   0.001   0.030
E Q  4GNKB               1156    0.539  96.230   0.821   0.970   0.001   0.030
E Q  4GNKB               1157    0.560  99.962   1.148   0.970   0.001   0.030
E V  4GNKB               1158    0.403  61.956   0.864   0.970   0.001   0.030
E L  4GNKB               1159    0.373  68.388   1.168   0.970   0.001   0.030
E Q  4GNKB               1160    0.595 106.267   1.456   0.970   0.001   0.030
E Q  4GNKB               1161    0.501  89.496   1.771   0.970   0.001   0.030
B L  4GNKB               1162    0.211  38.561   1.152   0.970   0.001   0.030
E A  4GNKB               1163    0.459  50.571   1.021   0.970   0.001   0.030
E E  4GNKB               1164    0.555  96.941   1.189   0.970   0.001   0.030
E E  4GNKB               1165    0.423  73.828   1.056   0.970   0.001   0.030
E E  4GNKB               1166    0.362  63.189   0.728   0.970   0.001   0.030
E P  4GNKB               1167    0.558  79.138   1.197   0.970   0.001   0.030
E K  4GNKB               1168    0.564 115.953   1.264   0.970   0.001   0.030
E L  4GNKB               1169    0.403  73.716   0.879   0.970   0.001   0.030
E L  4GNKB               1170    0.375  68.699   1.141   0.970   0.001   0.030
E A  4GNKB               1171    0.621  68.445   1.425   0.970   0.001   0.030
E Q  4GNKB               1172    0.528  94.283   1.516   0.970   0.001   0.030
B L  4GNKB               1173    0.256  46.819   1.254   0.970   0.001   0.030
E A  4GNKB               1174    0.525  57.844   1.155   0.970   0.001   0.030
E Q  4GNKB               1175    0.610 108.928   1.621   0.970   0.001   0.030
E E  4GNKB               1176    0.493  86.075   1.181   0.970   0.001   0.030
E C  4GNKB               1177    0.325  45.588   1.406   0.970   0.001   0.030
E Q  4GNKB               1178    0.589 105.267   1.473   0.970   0.001   0.030
E E  4GNKB               1179    0.582 101.710   1.387   0.970   0.001   0.030
E Q  4GNKB               1180    0.419  74.762   1.527   0.970   0.001   0.030
E R  4GNKB               1181    0.311  71.288   1.401   0.970   0.001   0.030
E A  4GNKB               1182    0.622  68.511   1.310   0.970   0.001   0.030
E R  4GNKB               1183    0.558 127.805   1.505   0.923   0.002   0.076
B L  4GNKB               1184    0.254  46.434   1.267   0.923   0.002   0.076
E P  4GNKB               1185    0.584  82.841   1.367   0.970   0.001   0.030
E Q  4GNKB               1186    0.565 100.963   1.340   0.970   0.001   0.030
E E  4GNKB               1187    0.464  81.008   1.172   0.975   0.003   0.022
B I  4GNKB               1188    0.262  48.470   0.729   0.975   0.003   0.022
E R  4GNKB               1189    0.568 130.164   1.543   0.970   0.001   0.030
E R  4GNKB               1190    0.567 129.912   1.414   0.970   0.001   0.030
E S  4GNKB               1191    0.416  48.732   1.077   0.970   0.001   0.030
E L  4GNKB               1192    0.347  63.609   0.426   0.970   0.001   0.030
E L  4GNKB               1193    0.550 100.687   1.168   0.970   0.001   0.030
E G  4GNKB               1194    0.636  50.037   1.291   0.970   0.001   0.030
E E  4GNKB               1195    0.377  65.897   0.827   0.970   0.001   0.030
E M  4GNKB               1196    0.401  80.240   0.080   0.923   0.002   0.076
E P  4GNKB               1197    0.593  84.218   0.657   0.923   0.002   0.076
E E  4GNKB               1198    0.575 100.522   0.793   0.970   0.001   0.030
E G  4GNKB               1199    0.420  33.046   0.121   0.970   0.001   0.030
E L  4GNKB               1200    0.418  76.609  -0.203   0.923   0.002   0.076
E G  4GNKB               1201    0.591  46.520   0.777   0.923   0.002   0.076
E D  4GNKB               1202    0.563  81.099   0.294   0.858   0.002   0.139
E G  4GNKB               1203    0.455  35.801   0.482   0.923   0.002   0.076
E P  4GNKB               1204    0.491  69.701   0.037   0.970   0.001   0.030
E L  4GNKB               1205    0.489  89.627   0.306   0.970   0.001   0.030
E V  4GNKB               1206    0.456  70.118   0.342   0.970   0.001   0.030
E A  4GNKB               1207    0.481  52.995   0.226   0.970   0.001   0.030
E C  4GNKB               1208    0.449  63.012   0.473   0.970   0.001   0.030
E A  4GNKB               1209    0.527  58.020   0.045   0.923   0.002   0.076
E S  4GNKB               1210    0.514  60.299  -0.011   0.923   0.002   0.076
E N  4GNKB               1211    0.527  77.109   0.597   0.858   0.002   0.139
E G  4GNKB               1212    0.559  44.033   0.712   0.858   0.002   0.139
E H  4GNKB               1213    0.453  82.383   0.140   0.858   0.002   0.139
E A  4GNKB               1214    0.475  52.356   0.452   0.923   0.002   0.076
E P  4GNKB               1215    0.578  82.075   0.610   0.970   0.001   0.030
E G  4GNKB               1216    0.479  37.713   0.244   0.970   0.001   0.030
E S  4GNKB               1217    0.474  55.576  -0.111   0.970   0.001   0.030
E S  4GNKB               1218    0.538  63.100   0.431   0.970   0.001   0.030
E G  4GNKB               1219    0.543  42.726   0.507   0.970   0.001   0.030
E H  4GNKB               1220    0.454  82.546   0.929   0.970   0.001   0.030
E L  4GNKB               1221    0.448  82.029   0.579   0.970   0.001   0.030
E S  4GNKB               1222    0.515  60.381   0.791   0.970   0.001   0.030
E G  4GNKB               1223    0.518  40.774   0.624   0.970   0.001   0.030
E A  4GNKB               1224    0.319  35.121   0.510   0.970   0.001   0.030
E D  4GNKB               1225    0.596  85.941   0.875   0.970   0.001   0.030
E S  4GNKB               1226    0.540  63.265   0.730   0.970   0.001   0.030
E E  4GNKB               1227    0.432  75.470   0.841   0.970   0.001   0.030
E S  4GNKB               1228    0.522  61.225   0.955   0.970   0.001   0.030
E Q  4GNKB               1229    0.540  96.408   1.047   0.970   0.001   0.030
E E  4GNKB               1230    0.499  87.228   0.948   0.923   0.002   0.076
E E  4GNKB               1231    0.496  86.634   0.475   0.923   0.002   0.076
E N  4GNKB               1232    0.547  80.081   0.274   0.858   0.002   0.139
E T  4GNKB               1233    0.671  93.109   0.947   0.600   0.003   0.397
E Q  4GNKB               1234    0.632 112.822   1.295   0.406   0.004   0.590
E L  4GNKB               1235    0.473  86.606  -0.883   0.003   0.003   0.994

Explain the output.  Go back.
```

---
